# Molecular Insights into the Dynamics of Pharmacogenetically Important N-Terminal Variants of the Human β_2_-Adrenergic Receptor

**DOI:** 10.1371/journal.pcbi.1004006

**Published:** 2014-12-11

**Authors:** Ganesh Shahane, Chirag Parsania, Durba Sengupta, Manali Joshi

**Affiliations:** 1 CSIR-National Chemical Laboratory, Pune, India; 2 Bioinformatics Center, University of Pune, Pune, India; University of Houston, United States of America

## Abstract

The human β_2_-adrenergic receptor (β_2_AR), a member of the G-protein coupled receptor (GPCR) family, is expressed in bronchial smooth muscle cells. Upon activation by agonists, β_2_AR causes bronchodilation and relief in asthma patients. The N-terminal polymorphism of β_2_AR at the 16^th^ position, Arg16Gly, has warranted a lot of attention since it is linked to variations in response to albuterol (agonist) treatment. Although the β_2_AR is one of the well-studied GPCRs, the N-terminus which harbors this mutation, is absent in all available experimental structures. The goal of this work was to study the molecular level differences between the N-terminal variants using structural modeling and atomistic molecular dynamics simulations. Our simulations reveal that the N-terminal region of the Arg variant shows greater dynamics than the Gly variant, leading to differential placement. Further, the position and dynamics of the N-terminal region, further, affects the ligand binding-site accessibility. Interestingly, long-range effects are also seen at the ligand binding site, which is marginally larger in the Gly as compared to the Arg variant resulting in the preferential docking of albuterol to the Gly variant. This study thus reveals key differences between the variants providing a molecular framework towards understanding the variable drug response in asthma patients.

## Author Summary

The human β_2_-adrenergic receptor (β_2_AR) is an important member of the GPCR family and a mutation at the 16^th^ position, Arg16Gly, is commonly found in the population. This variation in asthma patients is linked to differential (good/bad) response to the drug albuterol, an agonist of the β_2_AR. To date, the coordinates of the N-terminal residues harboring the 16^th^ position mutation have not been resolved. In our study we sought to glean insights into the dynamics of the variants that could address the differential response to albuterol. We used knowledge from class A GPCRs to build the N-terminal region of β_2_AR variants in conjunction with the available structure of the inactive receptor. This was followed by atomistic simulations in triplicate totaling to a sampling of 6 µs. We observe that the N-terminal region of the Arg variant is more dynamic than the Gly variant. Amongst the various differences between the variants, we observe long-range effects at the binding site leading to preferential docking of albuterol to the Gly variant. Our work is a first step to unravel the molecular mechanism linking the Arg16Gly variation to the differential response to albuterol in asthma patients.

## Introduction

G protein-coupled receptors (GPCRs) constitute a family of membrane proteins that serve as important communication mediators in cellular signal transduction [1], [2]. GPCRs are thus critical in the modulation of several signaling related disorders [3], [4] and constitute more than 25% of all human drug targets [5]. The human β_2_-adrenergic receptor (β_2_AR) is a member of the GPCR family that is abundantly distributed in smooth airway muscles of lung [6]. Endogenous catecholamine's such as epinephrine and norepinephrine act as agonists and bind β_2_AR causing smooth muscle relaxation and aiding respiration [7]. Agonists of β_2_AR such as albuterol, terbutaline (examples of short acting drugs), salmeterol and formeterol (long acting drugs), which cause respiratory smooth muscle relaxation are widely used in the treatment of asthma [8]. Agonist binding to β_2_AR triggers the activation of adenylyl cyclase via the G_s_ protein which leads to relaxation of the airway smooth muscles and relief from bronchospasm [9].

A number of genetic polymorphisms have been described in the gene (ADRB2) encoding for the β_2_AR [10]. The non-synonymous single nucleotide polymorphism (SNP) Arg16Gly is common in the population with a minor allele frequency of about 50% [10] and has been implicated in variable response to albuterol treatment [11], [12]. Clinical studies performed to investigate the association of this SNP with response to albuterol show results that vary between studies and across populations [13]. In a cell based assay, the 16^th^ position variants were found to be expressed at similar abundances, but displayed dissimilar kinetics upon repeated agonist treatment implicating the N-terminal region in receptor activation [14]. Further, slight differences in the binding affinity of epinephrine were found between the variants in competition binding studies [14]. Down regulation of β_2_AR is seen in response to chronic exposure to agonists [15], but is not likely to play a role in the differential drug response to albuterol which is used as a short acting drug. In summary, it was suggested that molecular level studies would help clarify the role of the N-terminal variants in albuterol binding and it is hypothesized that the differences in response to albuterol could arise due to varying dynamics of the N-terminal regions [14].

The β_2_AR is a 413 amino acid residue protein with three intracellular and three extracellular loops [16], [17]. The N-terminal region of the receptor is 28 residues long (as per β_2_AR crystal structures, where the 29^th^ amino acid is the first helical residue) [17]. Although several crystal structures of β_2_AR are now available in the public domain representing both active and inactive forms, the coordinates for the complete N-terminal region are missing in all the structures [16]–[23]. Various computational studies of β_2_AR have probed the mechanism of ligand entry, exit, binding and activation [24]–[27]. However, to the best of our knowledge, none of the studies have included the N-terminal region of β_2_AR.

It still remains unclear how a variation at the 16^th^ position might affect response to albuterol. The goal of this work was to explore the molecular mechanism that could lead to a differential response to albuterol. Towards this we modeled the N-terminal region and its variants at the 16^th^ position (Arg16Gly) in conjunction with the available structure of the inactive receptor. We further performed six (three each) unbiased atomistic molecular dynamics (MD) simulations of the Arg and Gly variants totaling to 6 µs. We analyzed the differences in the local and global conformational dynamics arising from the N-terminal variations. Our results are an important first step towards understanding the role of the polymorphism in the molecular mechanism of β_2_AR.

## Results

The N-terminal regions of GPCRs are difficult to resolve in structural experiments since they are highly dynamic and often contain tags for purification. The N-terminal region of the β_2_AR is as yet uncharacterized but harbors the pharmacogenetically relevant Arg16Gly mutation. We built a model of the N-terminal region in conjunction with the available structure of the inactive receptor. Subsequently, MD simulations were performed to glean molecular insights into the differences in the dynamics of the variants.

### Structural modeling of the pharmacogenetically relevant N-terminal variants

Structural models of the 16th position Arg and Gly variants of β_2_AR were generated as discussed in the methods section. Of all the class A GPCRs at the GPCRDB [28] more than thirty structures have complete or partial N-termini which are all placed on top of the seven transmembrane (TM) helices (S1 Table). Five class A GPCRs with N-termini of length similar to that of β_2_AR were chosen as templates for modeling. Although the sequence similarity of the β_2_AR with the templates in this region is poor, we chose to build our models based on available templates rather than *ab-initio* folding. Using related GPCR templates allows us to capture the structural characteristics of the family instead of the computationally daunting *ab initio* folding. Results from the Ramachandran plot analysis of the N-terminal regions indicate that for the Arg variant all the residues are in the allowed region while in Gly variant all but one residue are in the allowed region (S1 Figure).

**Figure 1:**
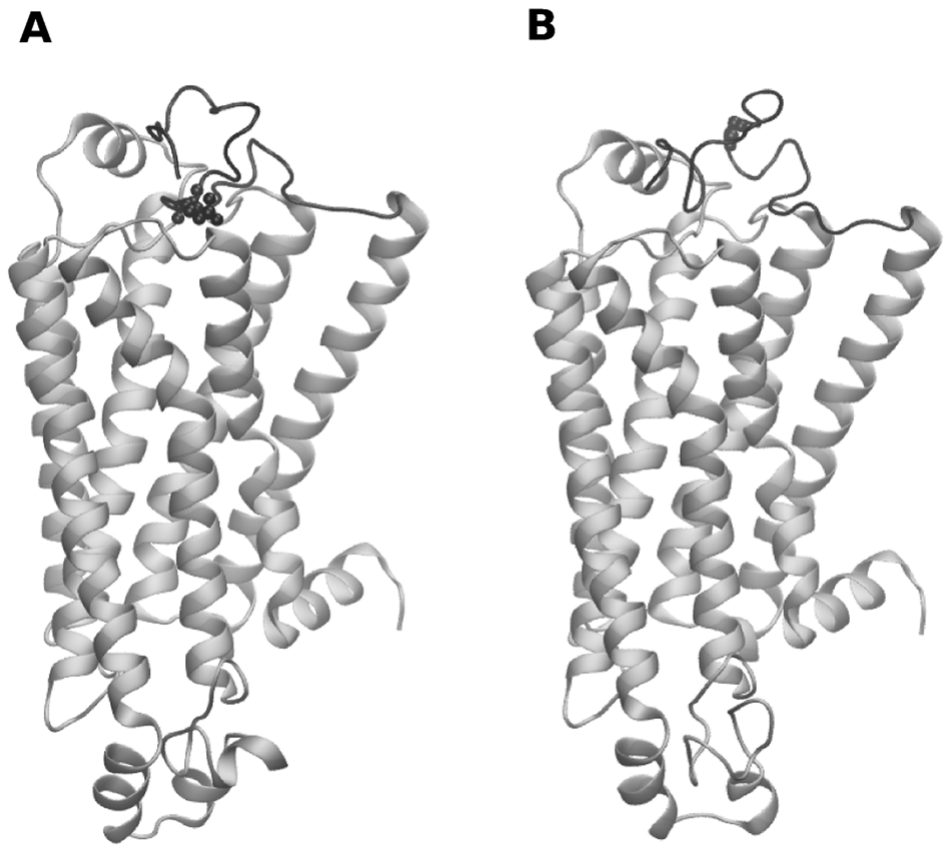
Schematic representation of the structural models of the β_2_AR variants. Panel A represents the Arg and panel B represents the Gly variants of β2AR, respectively. The N-terminal region is colored black and the rest of the receptor is colored silver. Residues at the 16^th^ position are displayed in the CPK representation.

In both the models, the placement of the N-terminal residues is on top of the receptor (Fig. 1). The overall secondary structure of the N-terminal region is mainly comprised of turns. In the Arg variant the N-terminal residues 5 to 13, 15 to 19 and 21 to 24 adopt a turn conformation as defined by STRIDE [29]. The N-terminal residues 5 to 8, 10 to 13 and 17 to 21 of the Gly variant adopt a turn conformation. The side chain of the arginine at the 16^th^ position is cradled within residues 301 to 305 from the TM region and its guanidinium group is placed in close proximity of Glu 306. In contrast, the glycine residue at that position is not predicted to interact with the TM region in the model (based on contacts within 0.5 nm of the residue).

### Structure and dynamics of the N-terminal variants

**Figure 2:**
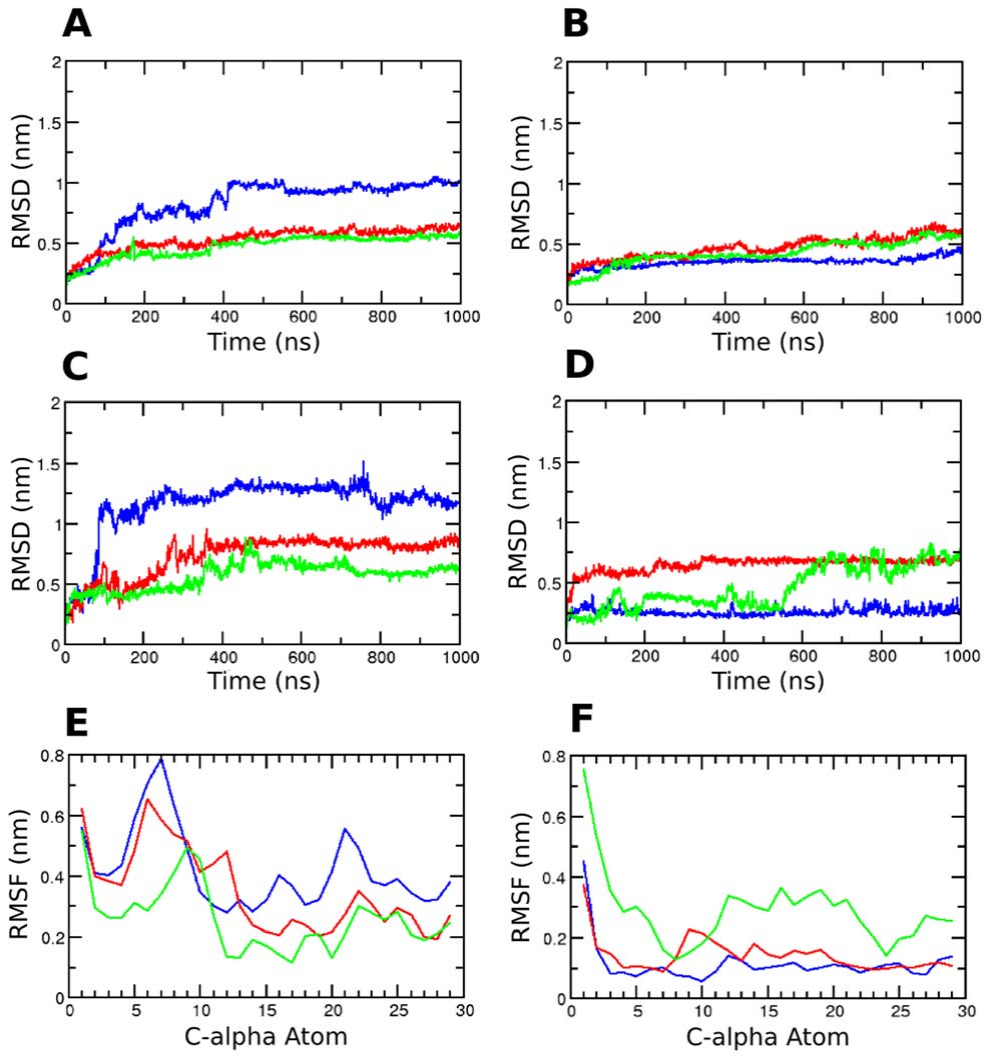
Structural characterization of the β_2_AR variants. All atom protein RMSD of (A) Arg and (B) Gly variants of β2AR with respect to the first frame of the production run. All atom RMSD of the N-terminal residues 1 to 29 of (C) Arg and (D) Gly variants of β2AR. RMSF profile of the N-terminal C-alpha atoms of (E) Arg and (F) Gly variants. For each plot, the blue line indicates the first simulation, the red indicates the second and the green line indicates the third simulation.

MD simulations of the variants of β_2_AR embedded in a lipid bilayer were performed in triplicate to ensure adequate sampling. To characterize the structural variation during the course of the simulation, the RMSD of the entire protein, for each of the six simulations was calculated (Fig. 2 A, B). The RMSD of the Arg variant, overall, is higher than the Gly variant. We further checked the RMSD of the TM helices, ICL3 and the N-terminal residues, separately to assess the contributions of the different regions of the receptor to the observed variation in RMSD for the entire protein. The TM helices were found to be stable over the simulation time (S2 Figure). Interestingly, the RMSD of the ICL3 is slightly higher in the simulations of the Arg variant as compared to the Gly variant (S2 Figure). The RMSD profiles of the N-terminal region (residue 1 to 28) reveal significant differences across the variants (Fig. 2 C, D). The three simulations of the Arg variant exhibit, on an average, a higher RMSD than the Gly variants suggesting enhanced dynamics. The large increase in the RMSD of the N-terminal region of the Arg variants contributes to the higher RMSD of the whole protein seen earlier. To understand the residue wise contribution of the N-terminal region to the conformational sampling we calculated the per-residue fluctuations over the simulations (Fig. 2 E, F). Consistent with the RMSD plots, the fluctuations of the residues in the Arg variants are generally higher. The first ten residues of the Arg variants show higher fluctuations than the corresponding residues in the Gly variant, specifically large differences are observed between residues 5–10.

**Figure 3:**
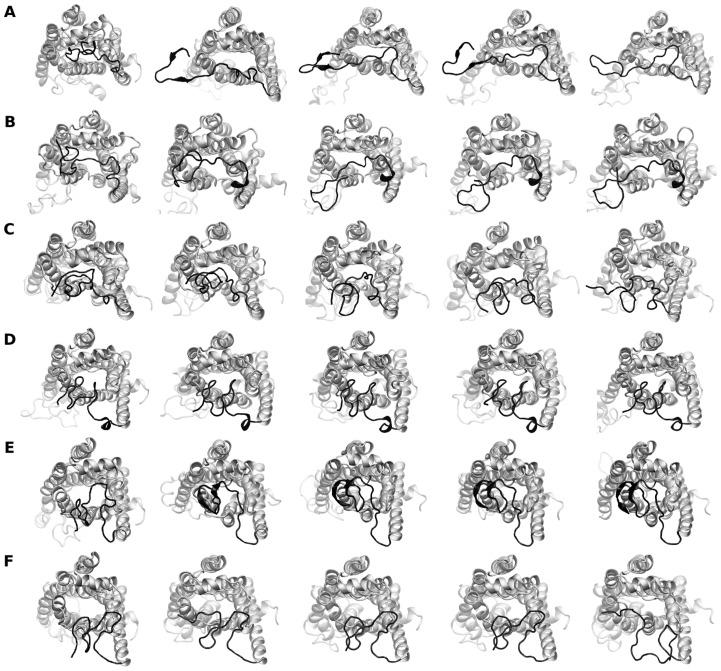
Top-view snapshots of the Arg and Gly variants of β_2_AR during the course of the simulation. The snapshots are shown at time intervals of 250 ns. Panel A, B and C represent simulation number 1, 2 and 3 of the Arg variant and panel D, E and F represent simulation number 1, 2 and 3 of the Gly variant, respectively. The protein is rendered as ribbons and the N-terminal residues are colored black.

To visualize the differences in the N-terminal region between the variants, representative snapshots of the protein from the trajectories were analyzed (Fig. 3). In all the three simulations of the Gly variants the N-terminal region is found to stay on top of the TM helices. In contrast, the Arg variants show larger dynamics and tend to open up partially.

**Figure 4:**
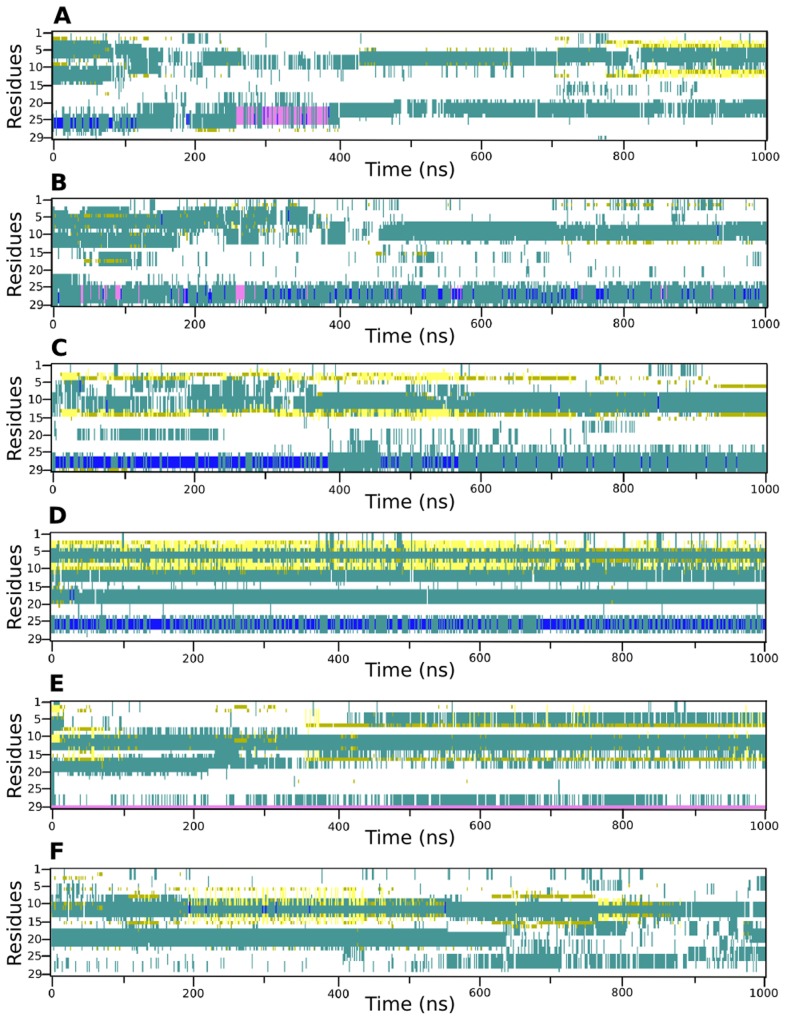
Secondary structure of the N-terminal region of the Arg and Gly variants of β_2_AR. Panels A, B and C represent simulation number 1, 2 and 3 of the Arg variant and panels D, E and F represent simulation number 1, 2 and 3 of the Gly variant, respectively. Turns are indicated by cyan color, 3–10 helices are indicated by blue, alpha helices are indicated by pink, isolated bridges are indicated by mustard and extended configuration is indicated by yellow color.

To quantify the structural differences at the N-terminal regions of the variants, the residue wise secondary structure was plotted over time (Fig. 4). In all three simulations of the Arg variant, after comprehensive sampling (400 ns onwards), a conformation comprising of two turns separated by nine to twelve residues is observed. The location of the turn varies between the simulations. The first turn is between residues 5–12 whereas the second is from 19–27. A turn that contains the 16^th^ position arginine, in the initial model opens up in all three simulations. On the other hand, in the three simulations of the Gly variant the two turns are consistently present between residues 10 to 13 and 17 to 19. In two simulations of the Gly variant, an additional turn is formed towards the N-terminal. The backbone of arginine at the 16th position in all three simulations of the Arg variant displays a lack of secondary structure character, whilst the glycine displays mainly a turn conformation. It is known that due to its small size, uncharged nature and thus unusual conformational ability, glycine is found in turns and can easily accommodate a turn in its vicinity.

**Figure 5:**
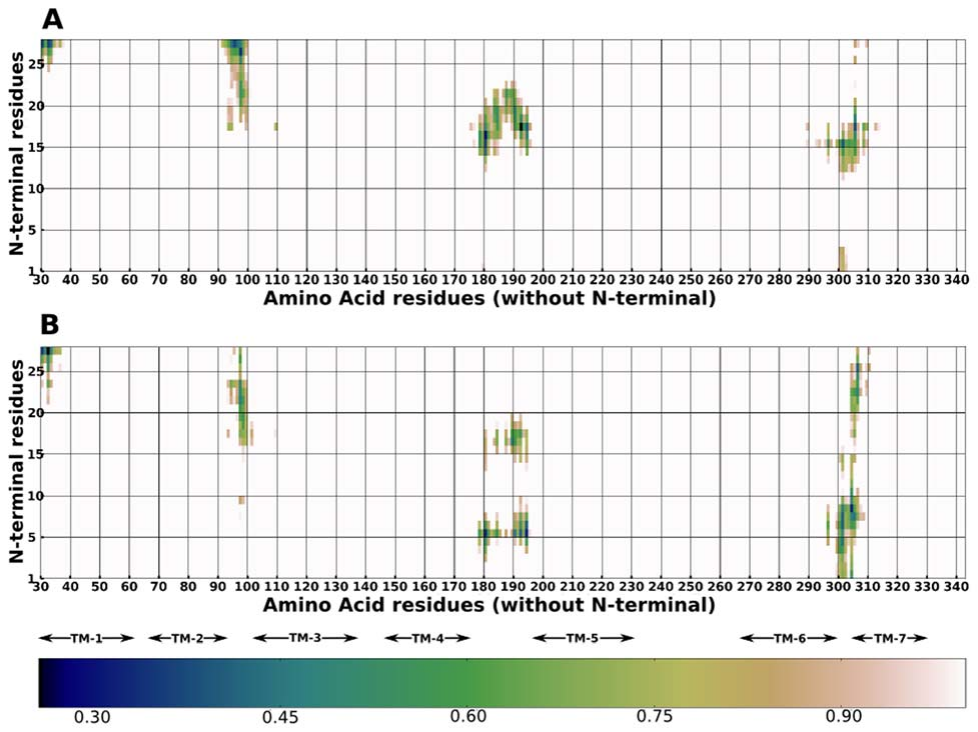
Contact maps of Arg and Gly variants of β_2_AR. Contacts between the N-terminal region of (A) Arg and (B) Gly variants and the rest of the receptor were computed from the average distance over simulation time. Average distances that were less than or equal to 1 nm were plotted. A color bar is indicated in which white indicates no-contacts and black indicates a close contact.

Further, side chain contacts of the N-terminal region with the receptor were calculated (Fig. 5). In addition, the contacts within 0.3 nm for at least 30% of the simulation time were calculated (S2 Table). A striking difference is seen in the number of contacts of the first fourteen residues of the variants. The Arg variant has very few contacts with the remaining receptor, while the residues of the Gly variant have several contacts as a result of the differential placement of the N-terminal region. Interestingly, the 16^th^ position arginine has several contacts with the receptor while the glycine at the 16^th^ position in the Gly variant has none. Further, residues 21 to 26 in the Gly variant interact with Glu 306 anchoring the N-terminal region to the 7^th^ helix. The residues 21 to 26 in the Arg variant on the other hand are closer to TM helix 2.

### Differences in the ligand binding pocket accessibility

β_2_AR binds water soluble ligands which are hypothesized to enter from the extracellular face of the receptor and further migrate inward to the actual binding pocket that is cradled in the transmembrane (TM) region [17]. Previous MD studies have demonstrated that ligand entry occurs via two possible pathways on the extracellular face of the receptor [24], [25], [30]. The entry to the two pathways is separated by a salt bridge between residues Asp 192 and Lys 305. The first pathway is on one side of the salt bridge and is formed by residues from TM helices 5, 6 and 7 (referred to as vestibule 1). The second pathway on the other side of the salt bridge comprises of residues from TM helices 2, 3 and 7 and is referred to as vestibule 2. Vestibule 1 is suggested to be the dominant pathway by both studies but they differed on the frequency of ligand entry via vestibule 2.

In the crystal structure (2RH1), the binding site cleft is entirely open and a salt bridge is seen to be formed between residues Asp 192 and Lys 305. The opening from vestibule 1 (defined in methods section) is seen to be much larger than that of vestibule 2. In the structural models that were built, the opening from vestibule 2 appears to be completely blocked while the opening from vestibule 1 stays partially open, due to the placement of the N-terminal residues on top of the receptor.

**Figure 6:**
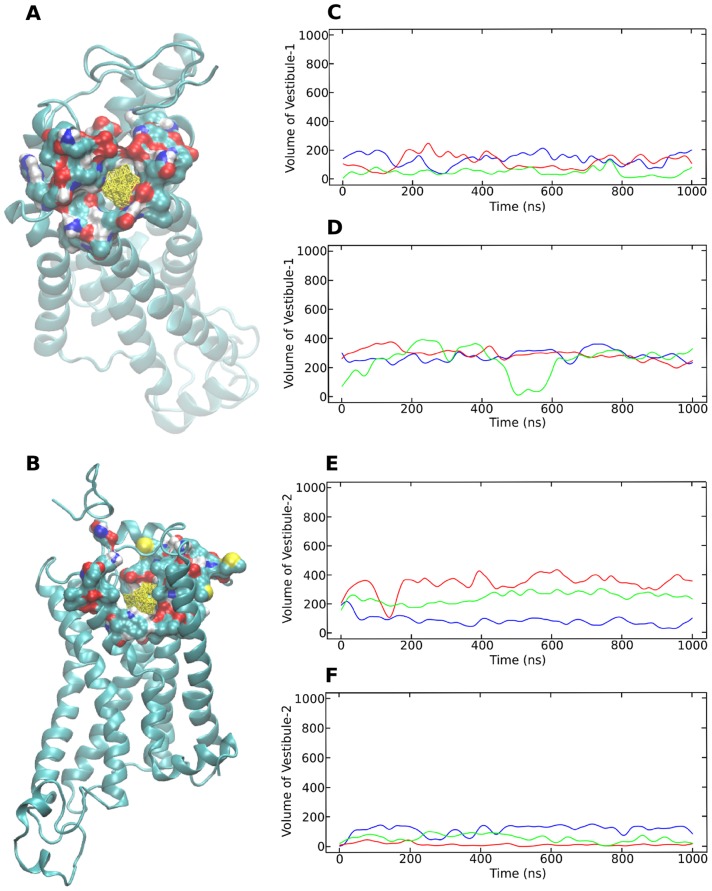
Vestibules of ligand entry in β_2_AR variants. Grids representing vestibule openings for (A) vestibule1 and (B) vestibule 2 of β_2_AR are shown in yellow. Residues defining the vestibules are shown in surface representation while the rest of the receptor is rendered as ribbons and colored blue. Panel C and D represent volumes (in Å^3^) of the non-occluded grid of vestibule 1 for the Arg and Gly variants, respectively. Panel E and F represent volumes (in Å^3^) of the non-occluded grid of vestibule 2 for the Arg and Gly variants, respectively. For each plot, the blue line indicates the first simulation, the red indicates the second and the green line indicates the third simulation.

The average volumes of the non-occluded grid points defining the vestibules was calculated and plotted against time (Fig. 6). Substantial fluctuations are observed in the volumes of the vestibule openings due to mobility of the side chains of the residues that line them. The plots indicate that the Gly variants on an average tend to have a more open vestibule 1 than their Arg counterparts. In the first 250 ns of the third simulation of the Gly variant, vestibule 1 opens up more due to lateral movement of the N-terminal region towards TM helix 6 and 7. The increased size of the vestibule is comparable to that observed in the crystal structure (2RH1). In the simulations of the Arg variants, the arginine residue at the 16th position interacts with residues lining vestibule 1 and in two out of three simulations partially blocks the vestibule with its bulky side chain. On the other hand, in the Gly variant the 16th position glycine is rarely seen to interact with the residues lining the vestibule, although residues 6 to 8 of the N-terminal region interact directly with these residues.

The reverse trend is seen at the site of vestibule 2. The Arg variants tend to have a more accessible vestibule 2 than the Gly variants, although there are large variations between the three simulations. The placement of the N-terminal region in the Gly variant in relation to the receptor, particularly the anchoring of the N-terminal region (residues 21 to 26) to the 7^th^ helix causes closure of the second vestibule. Since the N-terminal region stays on top of the receptor it leaves the first vestibule open. In the Arg variant, the N-terminal region (residues 21 to 26) is closer to helix 2 allowing the second vestibule to be open. In continuation, we tracked the salt bridge dividing the two vestibules. We observe that the Gly variant has a greater tendency to form a salt bridge as opposed to the Arg variant (S3 Figure).

To analyze whether the vestibules 1 and 2 differed from each other with respect to their electrostatic potentials, electrostatics were calculated using Delphi implemented in DS 3.5, for representative frames of the variants (S4 Figure). In the crystal structure, the potential around the binding site cleft is negative and was suggested to facilitate ligand entry through electrostatic funneling. The Gly variant tends to have a negative potential around vestibule 1 in contrast to the Arg variant. The difference can be attributed to the positive charge of the arginine at the 16^th^ position. In vestibule 2 of the Arg variant there is a large region at the entrance of the vestibule with a negative potential, however a small region of positive potential is also observed.

### Variations in the TM region of the receptor

**Figure 7:**
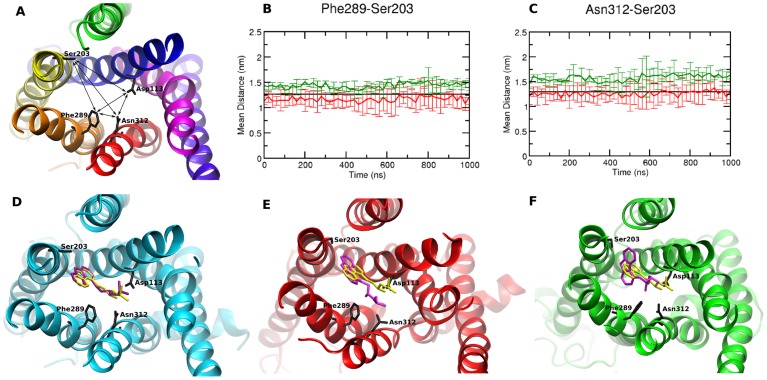
Characterization of the ligand binding site of β_2_AR variants. (A) Top-view of the β_2_AR represented as ribbons. The residues (113, 203, 289 and 312) that define the topology of the binding site are represented as licorice and the distances between them are indicated by lines. (B) Average distance between residues 289 and 203 for the Arg (red) and Gly (green) variants. (C) Average distance between residues 312 and 203 for the Arg (red) and Gly (green) variants. Docking of carazolol to (D) the 2RH1 structure, (E) Arg variant and (F) Gly variant. The crystal structure pose of carazolol is colored yellow, and the docked pose is colored purple.

The ligand binding pocket of β_2_AR is comprised primarily of residues belonging to TM helices 3, 6 and 7. Residues 113, 203, 289 and 312 define the topology of the binding pocket of β_2_AR (Fig. 7 A). Out of these, residues 113, 289 and 312 (3.32, 6.51 and 7.39) have been observed to make consensus contacts with various ligands in the class A GPCRs [31]. We measured the distances between these residues across the six simulations of the two variants. We observe that the average distances of the residue 203 with 312 and 289 are about 0.5 nm larger for the Gly variant than that for the Arg variant (Fig. 7 B, C). It thus appears that the overall binding pocket of the Gly variant in all three simulations is marginally larger than that of the Arg variant.

To determine the effects of the change in the binding pocket on ligand binding, we docked carazolol and albuterol to the pocket. Since the second simulation of the Arg variant shows the largest entry point from vestibule 2 and the first simulation of Gly shows the largest entry point from vestibule 1 we chose the last frames from these simulations for the docking calculations. We initially docked S-carazolol to the 2RH1 structure and observed that Glide-XP [32] was able to reproduce the binding mode (Fig. 7 D). We observed that the top ranking docking pose in the Arg variant is closer to the crystal structure pose than the top ranking pose (or any other) in the Gly variant. In the Arg variant the carbazole moiety is flipped by 180° as compared to the crystal structure pose, while in the Gly variant not only is the carbazole moiety tilted 90° in comparison to the crystal structure pose but also the polar side chain is at 90° in comparison to the crystal structure (Fig. 7 E, F). To test the stability of the predicted poses we performed short (50 ns) atomistic MD simulations on the complexes. Consistent with the docking studies, we observe that the carbazole moiety is stable in the Arg variant. In the Gly variant however, the carbazole moiety shows large dynamics and tilts between the different states (S5 Figure). The difference in binding mode of the carazolol to the Gly variant can be attributed to the larger binding site. The docking score of S-carazolol with the Gly variant (docking score −7.1) is comparable to that of the Arg variant (−7.9). Interestingly, the Gly variant has a much more favorable docking score for R and S Albuterol (−8.5, −9.5) as opposed to the Arg variant (−2.9,−5.7) (S6 Figure).

The ionic lock is a salt bridge formed between the two residues Arg 131 and Glu 268, from adjacent transmembrane helices 3 and 6. This salt bridge was implicated in the stabilization of the inactive state of GPCRs based on rhodopsin crystal structures. Consistent with previous studies [25], [33] on β2AR we observe the ionic lock to form occasionally in both the variants (S7 Figure). Overall, the Arg simulations showed an increased propensity to form the salt bridge. We further analyzed the consensus contacts as identified earlier in class A GPCRs [31] across the two variants and found that there are no significant differences except between residue 132 and 221. The contacts are indeed conserved across the two variants as they are in the class A GPCRs (S8 Figure).

## Discussion

GPCRs are important mediators in cellular signaling cascades and constitute a large percentage of current clinical drug targets [1]. The advent of next-generation sequencing tools has enabled the detection of polymorphisms in GPCRs that could be linked to disease and drug efficacy. The natural variant in β_2_AR at the 16th position has been implicated in a heterogeneous response to albuterol in asthma patients [10]–[13]. The N-terminal region of the β_2_AR that contains this variant is structurally unresolved [16]–[23]. The goal of our study was to provide a link between the variation in the structure and its functional implications. Towards the same we modeled the N-terminal region of the β_2_AR based on knowledge from related class A GPCRs and performed microsecond MD simulations of the receptor variants embedded in membranes.

We observe from our simulations that the N-terminal region of the Arg variant is more dynamic in contrast to the Gly variant that displays limited positional sampling. Further, the Arg variants tend to open up and residues 1 to 14 display no contacts with the rest of the receptor. Interestingly, a few of the β_2_AR crystal structures that contain an unresolved N-terminal region were expressed using gene constructs that code for Arg at the 16^th^ position[16], [17]. Due to the restrained dynamics that we observe in the Gly variant, it is possible that this variant would be a better candidate for crystallization studies focusing on the N-terminal region.

The difference at the N-terminal 16^th^ position affects ligand accessibility and binding through long-distance effects. In particular, the ligand binding site is more accessible through vestibule 1 of the Gly variant as opposed to vestibule 2 of the Arg variant. Previous computational studies of β_2_AR although lacking the N-terminal region that caps the β_2_AR in our models have demonstrated that ligand entry and exit via vestibule 1 was the lowest energy pathway [24], [25]. Thus, it seems that ligand entry that would occur via vestibule 2 in the Arg variant would be less favorable than ligand entry through vestibule 1 of the Gly variant. It has been previously proposed that differences in binding site accessibility could affect drug receptor kinetics [34]. The differences observed in the binding site accessibility in our simulations could thus affect β_2_AR activation kinetics by albuterol.

Structural plasticity in GPCRs has been suggested to be critical [35] and sub-nm scale differences in the binding pocket of GPCRs have been implicated in altered receptor function [36]. In our studies, the ligand binding pocket is marginally larger in the Gly variant and shows more favorable docking to albuterol. The effects induced by the N-terminal variants on the TM region in our simulations, although subtle, affect the functionally-relevant structural plasticity of β_2_AR. A conformational coupling of the extracellular region (extracellular loops 2 and 3) with ligand binding site has been previously observed by experiments [18]. A similar effect of the extracellular N-terminal region on the ligand-binding pocket is observed in our study. Thus, it is likely that the differential binding site size and accessibility between the variants could lead to the observed altered kinetics of albuterol in the cell based assay [14].

To check the statistical reliability of the major results of our study, we chose two additional models that differed maximally from the initial models in terms of RMSD of the N-terminal residues and performed 100 ns of atomistic simulations for each. In line with the previous results, the Arg variants showed a preference for a more accessible vestibule 2 while the Gly variants showed a preference for a more accessible vestibule 1 (S9 Figure) Further, the Gly variant showed a slightly larger distance between residues 203 and 289 although no differences were observed in the distance between residues 203 and 312. Thus despite the limited sampling in these models, they nevertheless validate the main findings of the study.

The influence of the lipid bilayer in GPCR structure and dynamics is currently being recognized as important [37]–[39]. Although, the N-terminal region modeled here does not directly interact with the lipid bilayer, we cannot rule out the possibility of indirect interactions of the N-terminal region with bilayer components in multi-component bilayers. In particular, negatively charged lipids have been implicated in influencing the conformational orientation of the juxtamembrane regions in receptor tyrosine kinases [40], and could play similar roles in the charged Arg variants of β_2_AR.

Our study is limited to microsecond time regimes and longer timescale studies might unveil further differences between the variants. Allosteric networks that lead to activation of β_2_AR have been demonstrated using microsecond time scale simulations [25], [41]. These networks involve the coupling of the extracellular loops 2 and 3 with the G protein binding site but the study did not include the N-terminal region. In light of the coupling seen in our simulation between the N-terminal region and the ligand binding site, it would be interesting to recalculate these allosteric networks including the N-terminal region of the two variants. It would be further interesting to simulate ligand entry into the variants, which has not been carried out in this study.

In conclusion, we probed the N-terminal polymorphism at the 16^th^ position of β_2_AR, Arg16Gly, which is linked to variations in response to albuterol treatment. The N-terminal region is unresolved in all experimental structures of β_2_AR. Using structural models of the N-terminal region of the variants in conjunction with the rest of the receptor followed by 6 µs of atomistic simulations, we are able to observe molecular level differences between the variants in the timescales of our simulations. Most notably, the N-terminal region of the Arg variant is more dynamic than that of the Gly variant. Positional differences of the N-terminal regions are seen to affect the accessibility of ligand entry sites. While vestibule 1 is accessible and open in the Gly variants, vestibule 2 is accessible in the Arg variants. Further, we observe that the binding pocket of the Gly variant is slightly larger than the Arg variant. This difference in binding site size translates to a better docking score of Albuterol to the Gly variant. The differences between the variants arise due to both the charged and the bulky nature of the Arg side-chain compared to that of Gly. The charged side-chain moiety of Arg allows contacts with suitable partners towards vestibule 1, causing the first fifteen residues of the N-terminal region to extend outwards from the receptor. The Gly variant on the other hand can accommodate a coil in the midst of the N-terminal region due to its small size and hence the first fifteen residues in this case remain coiled. We thus provide for the first time a molecular framework linking the differences in structural dynamics of the Arg16Gly variants to differences in binding albuterol, in microsecond timescale atomistic MD simulations. These results provide new insights towards understanding the variable response in asthma patients.

## Methods

### Modeling β_2_AR variants

The primary sequence of human β_2_AR was retrieved from UniProt (Accession number P07550) and edited to generate the variant sequence. The crystal structure PDB ID:2RH1 was chosen as the reference structure for the coordinates from residue 29 to 342. For the N-terminal structure prediction, five class A GPCR templates were chosen (1U19, 2ZIY, 2KS9, 2L87, 2K03) whose N-terminal region had a similar length to that of β_2_AR (Table S3). In addition, the third intracellular loop (residues 231 to 262) was modeled using the templates 1U19, 2ZIY and 2KS9 since it is absent in the 2RH1 structure. Models were built using the MODELER [42] program (version 9.7) as implemented in Discovery Studio version 3.5 [43]. Fifteen models were generated for each variant of which three models including the one with the best energetics were chosen for further analysis.

### Molecular dynamics simulations

The structural models were taken as the initial structure for the simulations. The protonation state of ionisable residues was chosen as appropriate for pH 7.0 except for two residues, Glu 122 and His 172 which were protonated in accordance with the previous study of Dror *et al.*, [25]. The receptor was embedded in a fully hydrated POPC (1-palmitoyl-2oleoyl-*sn*-glycero-3-phosphocholine) bilayer (256 lipids). GROMOS54a7 force field was used to represent the protein [44] and a compatible force-field was used to represent the lipid [45]. The Simple Point Charge (SPC) model was used to represent the water [46]. Counter ions were added to make the system neutral by replacing water molecules.

The system was minimized and equilibrated. 100 ps NVT equilibration was followed by 25 ns NPT equilibration. All simulations were performed using the GROMACS version 4.5.5 package [47]. Periodic boundary conditions were applied. The system components were separately coupled to a temperature bath at 300 K with a coupling time constant of 0.5 ps [46]. For the short-range van der Waals and electrostatic cutoff, a distance of 1.2 nm was used. Long-range electrostatic interactions were calculated using the Particle-Mesh Ewald (PME) method [48]. Semi-isotropic pressure coupling was carried out using a Berendsen Barostat and the volume compressibility was chosen to be 4.5×10^−5^ bar^−1^ [46]. Three simulations of 1 µs each were performed for one of the Arg and Gly variant models and 100 ns simulations were performed for the remaining models. The structures were saved at a frequency of 1 ns.

### Analysis

All analysis was performed using standard GROMACS [47] and VMD tools [49]. For the RMSD calculation, the protein structures were aligned to the first frame of the simulation with respect to the particular region for which the RMSD was being measured. The secondary structure was calculated using the STRIDE program as implemented in VMD.

MDpocket [50] was used for detecting pockets along the course of the simulation. The algorithm is based on the principle of Voronoi tessellation. Residues 175–182, 192–200, 296–302 and 305 were chosen to define the opening of vestibule 1. Residues 86–99, 106–107, 109, 113, 189–192, 305–309 and 313 were chosen to define the opening of vestibule 2. In the first step, all six trajectories were superimposed based on vestibule 1 or 2 separately and a grid was placed in voids between the residues defining the vestibules. The grid points best representing the opening of vestibule 1 and 2 were hand edited and saved. All six trajectories aligned by entire length along with the edited grid were submitted for the final calculation. The vestibule volume was calculated from the non-occluded grid points.

### Docking

Docking studies of the ligands albuterol and carazolol to the variants were performed using GLIDE-XP [32]. Albuterol and carazolol were saved in the SMILES format from Pubchem [51] and were prepared using the Ligprep module of Maestro version 9.4 [52]. Although both the R and S chiral forms of carazolol were generated only the S form was analyzed to match clinical use and crystallization studies (2RH1 contains carazolol in the S form). Furthermore, only the +1 protonation state of the aliphatic amine was considered. The β_2_AR frames chosen for docking were prepared using the Protein preparation wizard in Maestro. Residues that were within 0.7 nm of carazolol in the 2RH1 structure were used to define the centroid of the grid for docking in the corresponding variants. A grid was generated that encompassed the above mentioned residues for docking. The prepared ligands were docked into the β_2_AR binding site and their binding modes were analyzed. The docked structures were further simulated for 50 ns using the protocol of the unliganded receptor. Parameters for S-carazolol were generated and obtained from ATB [53].

## Supporting Information

S1 Figure **Structural validation of the N-terminal region of the β_2_AR variant models.** Ramachandran plot analysis of the N-terminal region of (A) Arg and (B) Gly variants. The amino acid Gly is represented by triangles while Pro is represented as squares, the remaining residues are represented as circles.
(PDF)

S2 Figure **Structural characterization of the TM and ICL3 region of the β_2_AR variants.** All atom RMSD of the TM helices of the (A) Arg and (B) Gly variants. All atom RMSD of the ICL3 of (C) Arg and (D) Gly variants. Blue line indicates the first simulation, red line indicates the second simulation and the green line indicates the third simulation.
(PDF)

S3 Figure **Salt bridge separating the ligand entryways in the β_2_AR variants.** Distances between side-chains of Asp192 and Lys 305 in (A) Arg and (B) Gly variants. The blue lines indicate the first simulation, red lines indicate the second simulation and the green lines indicate the third simulation of each variant, respectively. The black line indicates the minimum distance defining the salt bridge.
(PDF)

S4 Figure **Electrostatic potential maps of the vestibules in the β_2_AR variants.** Electrostatic potential maps of (A) vestibule 2 of Arg and (B) vestibule 1 of the Gly variant. Electrostatics were calculated using Delphi implemented in DS 3.5 for representative frames of the variants.
(PDF)

S5 Figure **Interaction of S-carazolol with the β_2_AR variants.** A) Arg variant of β_2_AR with the docked pose of S-carazolol (magenta) and the pose after 50 ns simulation (yellow). B) Gly variant of β_2_AR with the docked pose of S-carazolol (magenta) and the pose after 50 ns simulation (yellow). C) RMSD of S-carazolol with respect to the first frame of the production run. Red line indicates Arg variant while the green line indicates the Gly variant. For the RMSD calculation the TM helices of subsequent frames were aligned to the first frame and the RMSD of carazolol with respect to the initial pose was calculated.
(PDF)

S6 Figure **Interaction of R and S-albuterol with the β_2_AR variants.** Docking of R-albuterol to (A) Arg variant and (B) Gly variant and the docking of S-albuterol to (C) Arg variant and (D) Gly variant. The protein is rendered as ribbons while the ligand is rendered as licorice and colored magenta.
(PDF)

S7 Figure **Characterization of the ionic lock in the β_2_AR variants.** Distances between side-chains of Glu268 and Arg131 in (A) Arg and (B) Gly variants. The blue lines indicate the first simulation, red lines indicate the second simulation and the green lines indicate the third simulation of each variant, respectively.
(PDF)

S8 Figure **Analysis of the consensus contacts as seen in class A GPCRs.** Distance between the side chains of the residue pairs (A) Ile47 & Gly320, (B) Gly50 & Pro323 (C) Asn51 & Ser319 (D) Val54 & Asn51 (E) Ile58 & Thr73 (F) Phe71 & Ile127 (G) Ile72 & Tyr326 (H) Ala76 & Val54 (I) Asp79 & Asn51 (J) Asp79 & Ser319 (K) Leu115 & Ser161 (L) Leu115 & Ser165 (M) Val117 & Met279 (N) Ala119 & Trp158 (O) Ala119 & Ser161 (P) Ile121 & Leu275 (Q) Cys125 & Met215 (R) Ala128 & Val218 (S) Tyr132 & Val218 (T) Tyr132 & Arg221 (U) Met215& Lys273 (V) Ile278 & Asn318 (W) Phe282 & Leu311 (X) Phe282 & Asn312. In each panel the red line indicates the distance for the Arg variant and the green line indicates the distance for the Gly variant respectively.
(PDF)

S9 Figure **Characterization of additional homology models of the β_2_AR variants.** Top-view snapshots of initial A) Arg model 2 B) Arg Model 3 C) Gly Model 2 D) Gly Model 3 chosen for 100 ns simulation. Panel E and F represent volumes (in Å^3^) of the non-occluded grid of vestibule 1 for the Arg and Gly variants, respectively. Panel G and H represent volumes (in Å^3^) of the non-occluded grid of vestibule 2 for the Arg and Gly variants, respectively. (I) Average distance between residues 289 and 203 for the Arg (red) and Gly (green) variants. (J) Average distance between residues 312 and 203 for the Arg (red) and Gly (green) variants.
(PDF)

S1 Table **Class A GPCRs from the GPCRDB which include full/partial N-terminal coordinates.**
(PDF)

S2 Table **Contacts of the N-terminal residues with the rest of the receptor that are within 0.3 nm for at least 30% of simulation time.**
(PDF)

S3 Table **Templates used to model the N-terminal region of human β2AR variants.**
(PDF)
