# Supplementary material for: Molecular Insights into the Dynamics of Pharmacogenetically Important N-Terminal Variants of the Human β2-Adrenergic Receptor
Source: PLoS Comput Biol. 2014 Dec 11;10(12):e1004006. doi: 10.1371/journal.pcbi.1004006 (PMC4263363; doi:10.1371/journal.pcbi.1004006)
Supplement: S1 Figure — Structural validation of the N-terminal region of the β2AR variant models. Ramachandran plot analysis of the N-terminal region of (A) Arg and (B) Gly variants. The amino acid Gly is represented by triangles while Pro is represented as squares, the remaining residues are represented as circles. (PDF) [file pcbi.1004006.s001.pdf]

A **B**

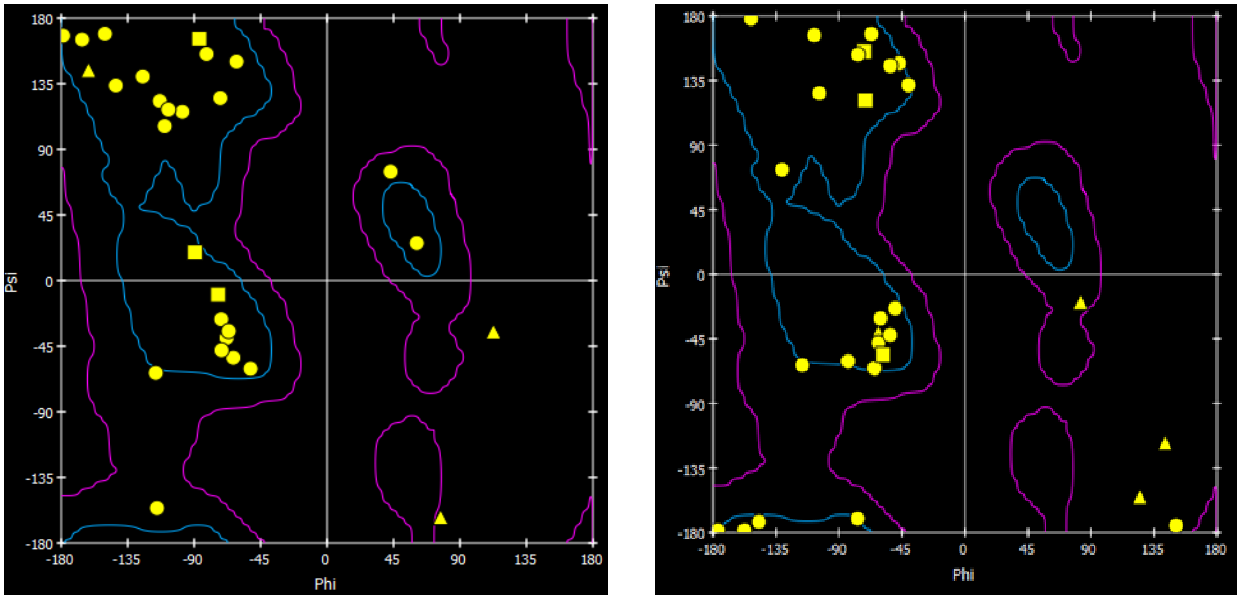

Supplementary Fig. 1: Ramachandran plot analysis of the N-terminal region of (A) Arg and (B) Gly variants. The amino acid Gly is represented by triangles while Pro is represented as squares, the remaining residues are represented as circles.
