# Supplementary material for: Molecular Insights into the Dynamics of Pharmacogenetically Important N-Terminal Variants of the Human β2-Adrenergic Receptor
Source: PLoS Comput Biol. 2014 Dec 11;10(12):e1004006. doi: 10.1371/journal.pcbi.1004006 (PMC4263363; doi:10.1371/journal.pcbi.1004006)
Supplement: S2 Figure — Structural characterization of the TM and ICL3 region of the β2AR variants. All atom RMSD of the TM helices of the (A) Arg and (B) Gly variants. All atom RMSD of the ICL3 of (C) Arg and (D) Gly variants. Blue line indicates the first simulation, red line indicates the second simulation and the green line indicates the third simulation. (PDF) [file pcbi.1004006.s002.pdf]

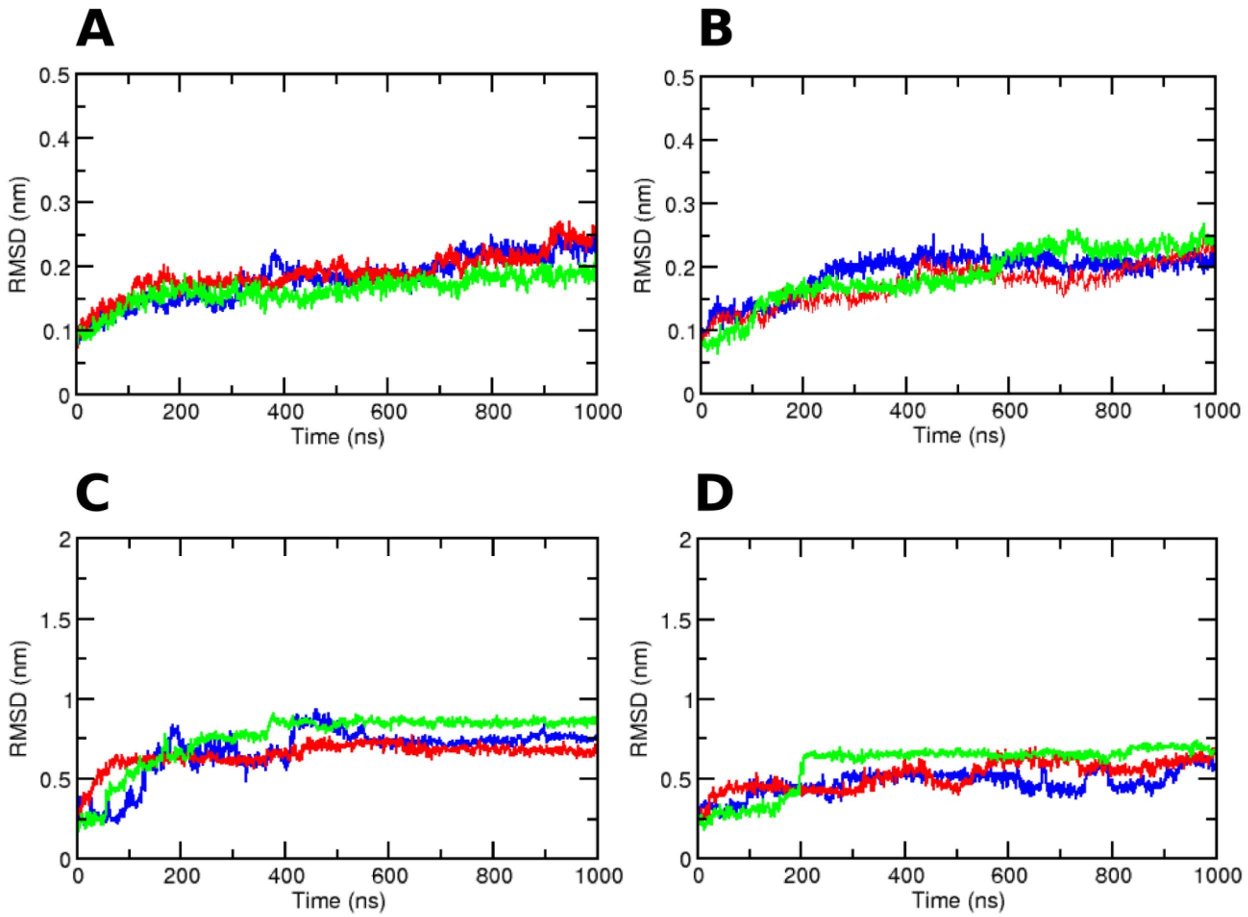

Supplementary Fig. 2: All atom RMSD of the TM helices of the (A) Arg and (B) Gly variants. All atom RMSD of the ICL3 of (C) Arg and (D) Gly variants. Blue line indicates the first simulation, red line indicates the second simulation and the green line indicates the third simulation
