# Supplementary material for: Molecular Insights into the Dynamics of Pharmacogenetically Important N-Terminal Variants of the Human β2-Adrenergic Receptor
Source: PLoS Comput Biol. 2014 Dec 11;10(12):e1004006. doi: 10.1371/journal.pcbi.1004006 (PMC4263363; doi:10.1371/journal.pcbi.1004006)
Supplement: S3 Figure — Salt bridge separating the ligand entryways in the β2AR variants. Distances between side-chains of Asp192 and Lys 305 in (A) Arg and (B) Gly variants. The blue lines indicate the first simulation, red lines indicate the second simulation and the green lines indicate the third simulation of each variant, respectively. The black line indicates the minimum distance defining the salt bridge. (PDF) [file pcbi.1004006.s003.pdf]

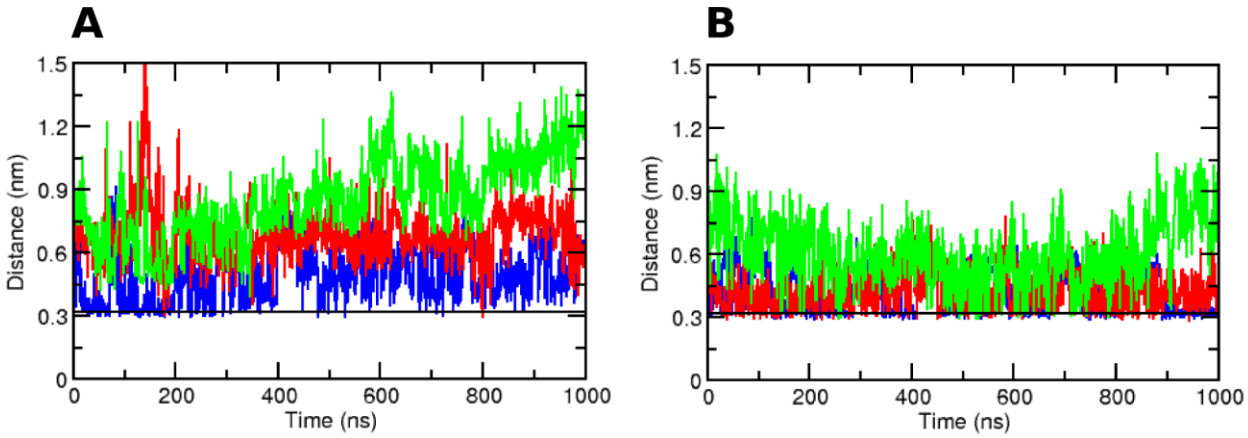

Supplementary Fig. 3: Distances between side-chains of Asp192 and Lys 305 in (A) Arg and (B) Gly variants. The blue lines indicate the first simulation, red lines indicate the second simulation and the green lines indicate the third simulation of each variant, respectively. The black line indicates the minimum distance defining the salt bridge.
