# Supplementary material for: Molecular Insights into the Dynamics of Pharmacogenetically Important N-Terminal Variants of the Human β2-Adrenergic Receptor
Source: PLoS Comput Biol. 2014 Dec 11;10(12):e1004006. doi: 10.1371/journal.pcbi.1004006 (PMC4263363; doi:10.1371/journal.pcbi.1004006)
Supplement: S4 Figure — Electrostatic potential maps of the vestibules in the β2AR variants. Electrostatic potential maps of (A) vestibule 2 of Arg and (B) vestibule 1 of the Gly variant. Electrostatics were calculated using Delphi implemented in DS 3.5 for representative frames of the variants. (PDF) [file pcbi.1004006.s004.pdf]

A **B**

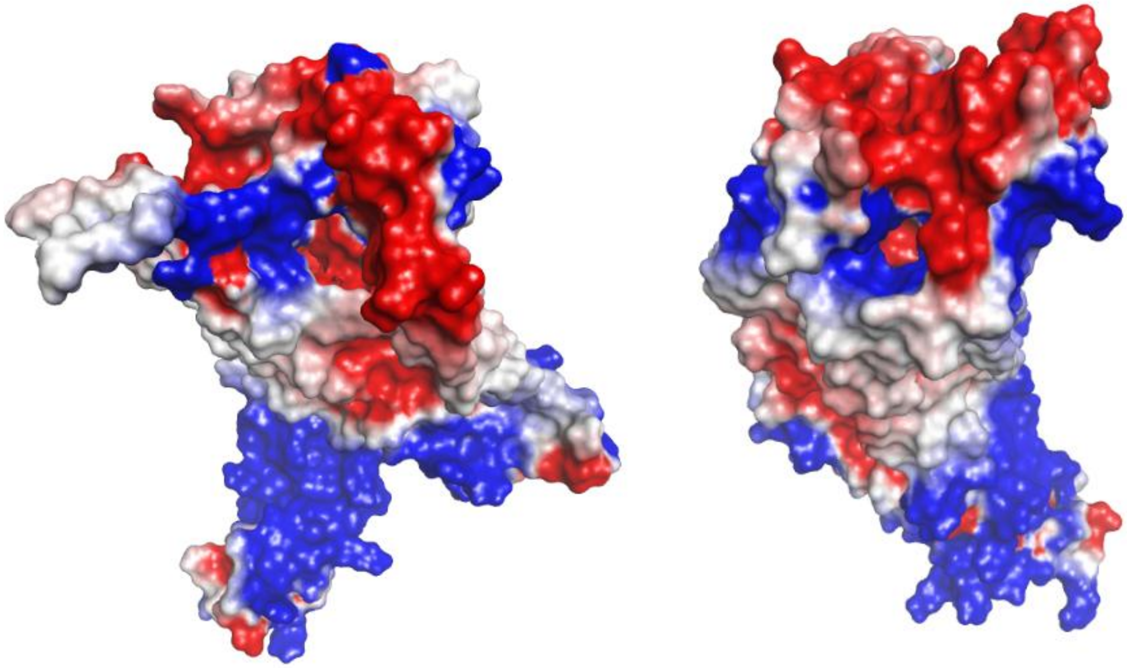

Supplementary Fig. 4: Electrostatic potential maps of (A) vestibule 2 of Arg and (B) vestibule 1 of the Gly variant. Electrostatics were calculated using Delphi implemented in DS 3.5 for representative frames of the variants.
