# Supplementary material for: Molecular Insights into the Dynamics of Pharmacogenetically Important N-Terminal Variants of the Human β2-Adrenergic Receptor
Source: PLoS Comput Biol. 2014 Dec 11;10(12):e1004006. doi: 10.1371/journal.pcbi.1004006 (PMC4263363; doi:10.1371/journal.pcbi.1004006)
Supplement: S5 Figure — Interaction of S-carazolol with the β2AR variants. A) Arg variant of β2AR with the docked pose of S-carazolol (magenta) and the pose after 50 ns simulation (yellow). B) Gly variant of β2AR with the docked pose of S-carazolol (magenta) and the pose after 50 ns simulation (yellow). C) RMSD of S-carazolol with respect to the first frame of the production run. Red line indicates Arg variant while the green line indicates the Gly variant. For the RMSD calculation the TM helices of subsequent frames were aligned to the first frame and the RMSD of carazolol with respect to the initial pose was calculated. (PDF) [file pcbi.1004006.s005.pdf]

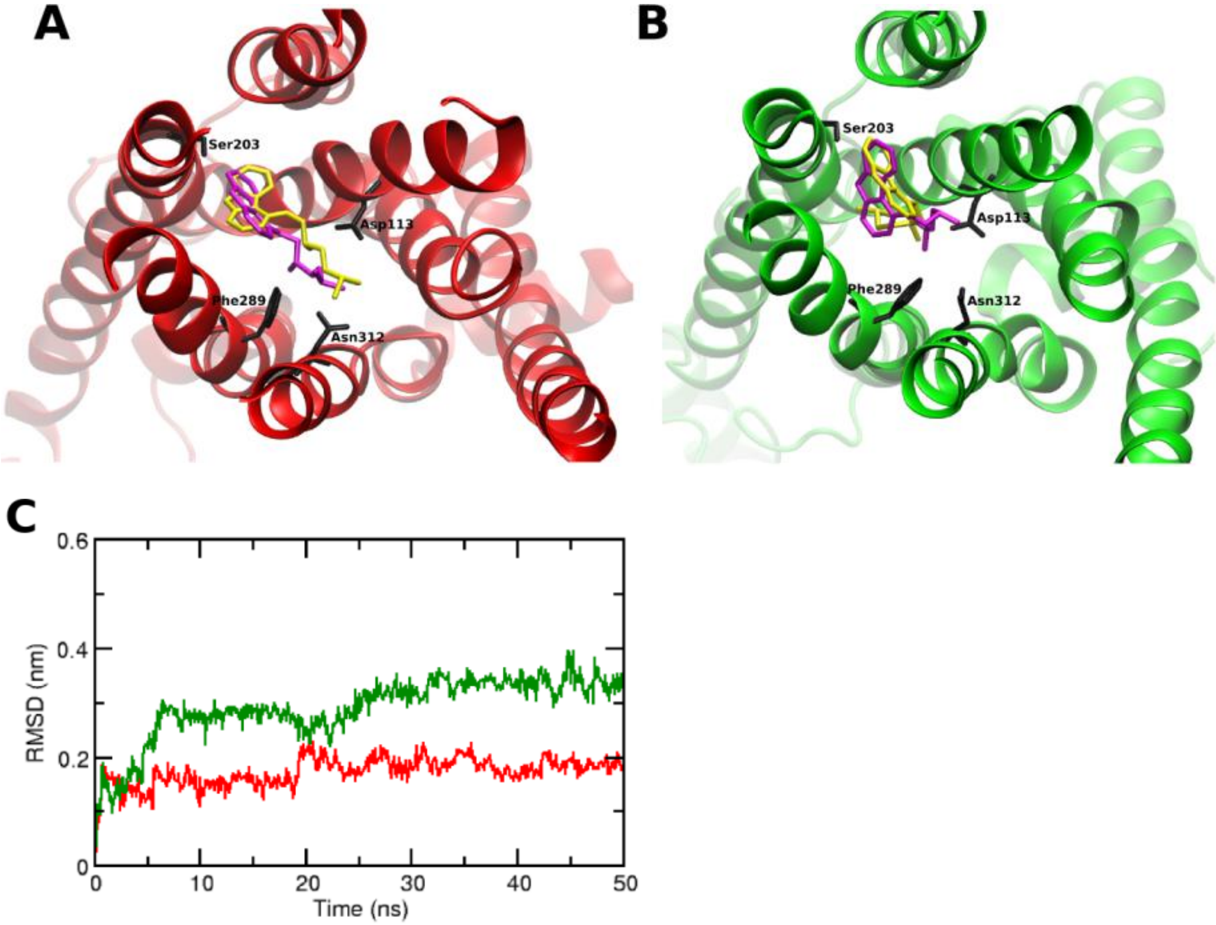

Supplementary Fig. 5: A) Arg variant of β$_2$AR with the docked pose of S-carazolol (magenta) and the pose after 50ns simulation (yellow). B) Gly variant of β$_2$AR with the docked pose of S-carazolol (magenta) and the pose after 50ns simulation (yellow). C) RMSD of S-carazolol with respect to the first frame of the production run. Red line indicates Arg variant while the green line indicates the Gly variant. For the RMSD calculation the TM helices of subsequent frames were aligned to the first frame and the RMSD of carazolol with respect to the initial pose was calculated.
