# Supplementary material for: Molecular Insights into the Dynamics of Pharmacogenetically Important N-Terminal Variants of the Human β2-Adrenergic Receptor
Source: PLoS Comput Biol. 2014 Dec 11;10(12):e1004006. doi: 10.1371/journal.pcbi.1004006 (PMC4263363; doi:10.1371/journal.pcbi.1004006)
Supplement: S6 Figure — Interaction of R and S-albuterol with the β2AR variants. Docking of R-albuterol to (A) Arg variant and (B) Gly variant and the docking of S-albuterol to (C) Arg variant and (D) Gly variant. The protein is rendered as ribbons while the ligand is rendered as licorice and colored magenta. (PDF) [file pcbi.1004006.s006.pdf]

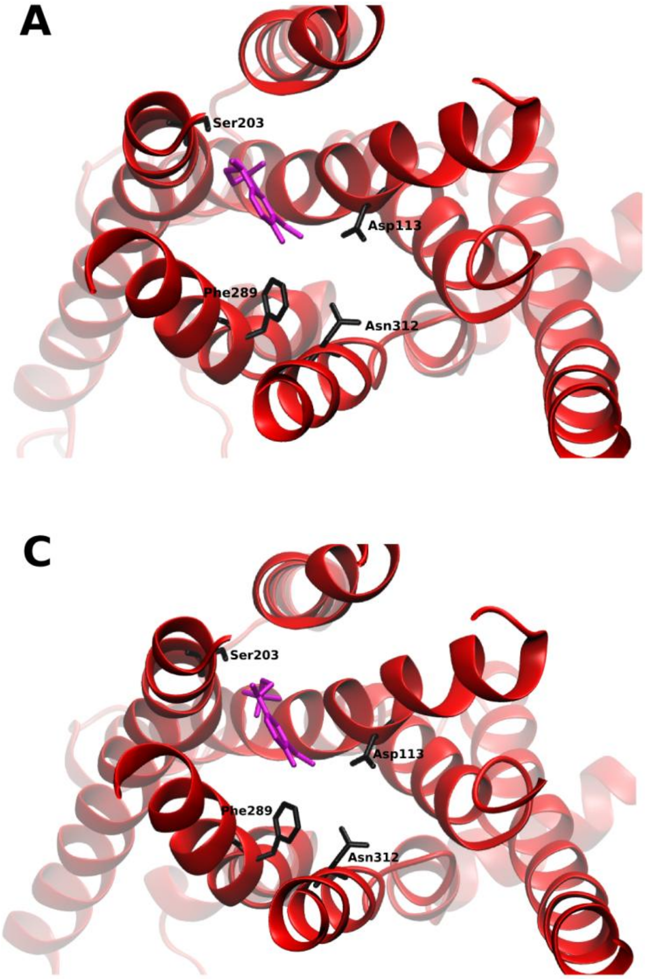

Supplementary Fig. 6: Docking of R-albuterol to (A) Arg variant and (B) Gly variant and the docking of S-albuterol to (C) Arg variant and (D) Gly variant. The protein is rendered as ribbons while the ligand is rendered as licorice and colored magenta.
