# Supplementary material for: Molecular Insights into the Dynamics of Pharmacogenetically Important N-Terminal Variants of the Human β2-Adrenergic Receptor
Source: PLoS Comput Biol. 2014 Dec 11;10(12):e1004006. doi: 10.1371/journal.pcbi.1004006 (PMC4263363; doi:10.1371/journal.pcbi.1004006)
Supplement: S7 Figure — Characterization of the ionic lock in the β2AR variants. Distances between side-chains of Glu268 and Arg131 in (A) Arg and (B) Gly variants. The blue lines indicate the first simulation, red lines indicate the second simulation and the green lines indicate the third simulation of each variant, respectively. (PDF) [file pcbi.1004006.s007.pdf]

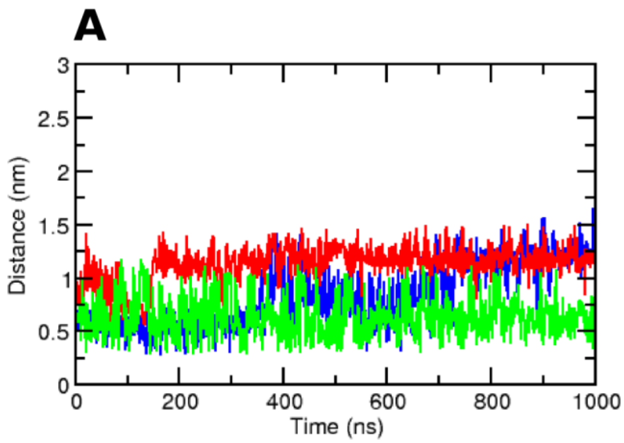

Supplementary Fig. 7: Distances between side-chains of Glu268 and Arg131 in (A) Arg and (B) Gly variants. The blue lines indicate the first simulation, red lines indicate the second simulation and the green lines indicate the third simulation of each variant, respectively.
