# Supplementary material for: Molecular Insights into the Dynamics of Pharmacogenetically Important N-Terminal Variants of the Human β2-Adrenergic Receptor
Source: PLoS Comput Biol. 2014 Dec 11;10(12):e1004006. doi: 10.1371/journal.pcbi.1004006 (PMC4263363; doi:10.1371/journal.pcbi.1004006)
Supplement: S8 Figure — Analysis of the consensus contacts as seen in class A GPCRs. Distance between the side chains of the residue pairs (A) Ile47 & Gly320, (B) Gly50 & Pro323 (C) Asn51 & Ser319 (D) Val54 & Asn51 (E) Ile58 & Thr73 (F) Phe71 & Ile127 (G) Ile72 & Tyr326 (H) Ala76 & Val54 (I) Asp79 & Asn51 (J) Asp79 & Ser319 (K) Leu115 & Ser161 (L) Leu115 & Ser165 (M) Val117 & Met279 (N) Ala119 & Trp158 (O) Ala119 & Ser161 (P) Ile121 & Leu275 (Q) Cys125 & Met215 (R) Ala128 & Val218 (S) Tyr132 & Val218 (T) Tyr132 & Arg221 (U) Met215& Lys273 (V) Ile278 & Asn318 (W) Phe282 & Leu311 (X) Phe282 & Asn312. In each panel the red line indicates the distance for the Arg variant and the green line indicates the distance for the Gly variant respectively. (PDF) [file pcbi.1004006.s008.pdf]

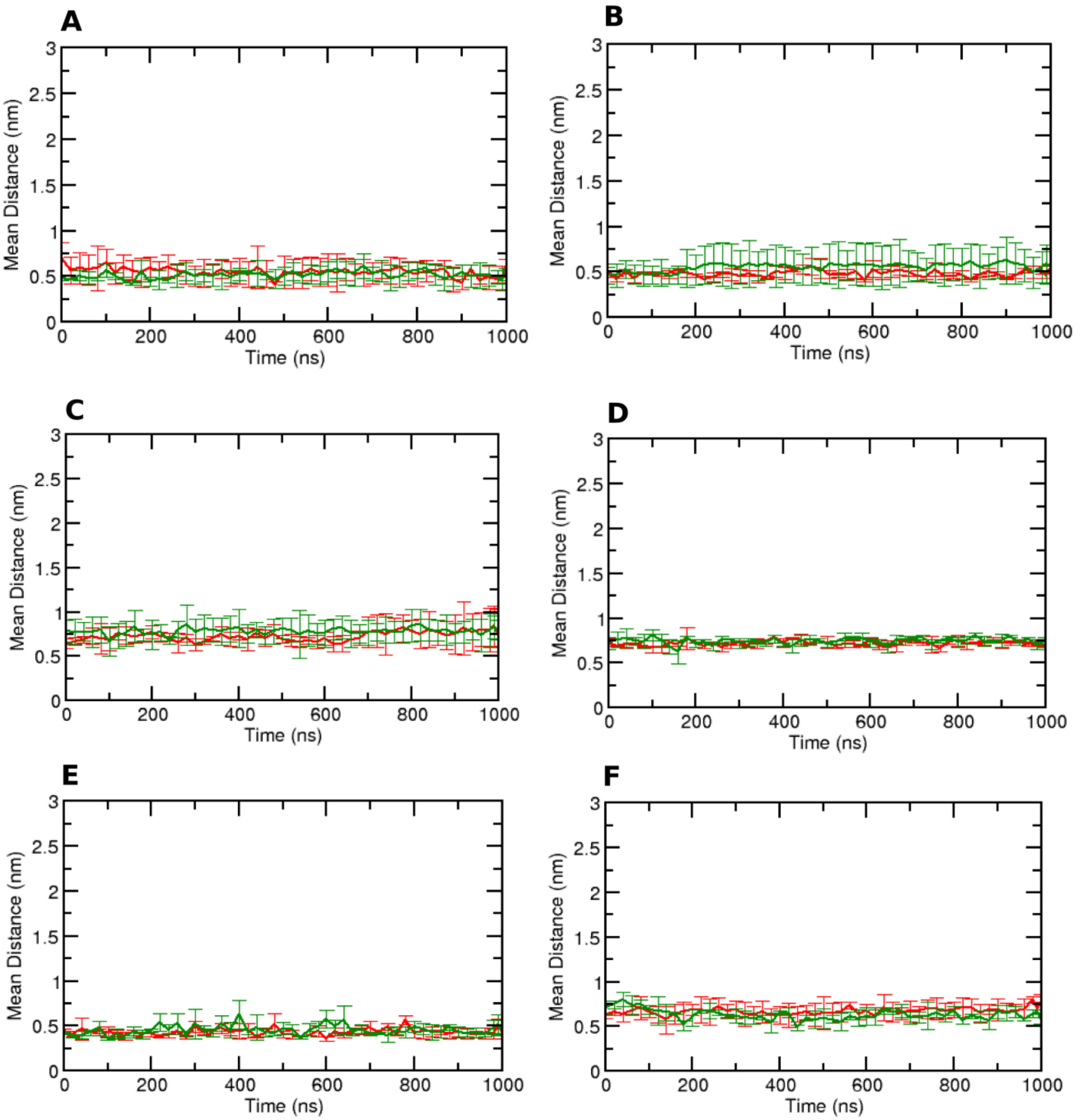

Shahane et al, Molecular insights into the dynamics of pharmacogenetically important N-terminal variants of the human β_2-adrenergic receptor

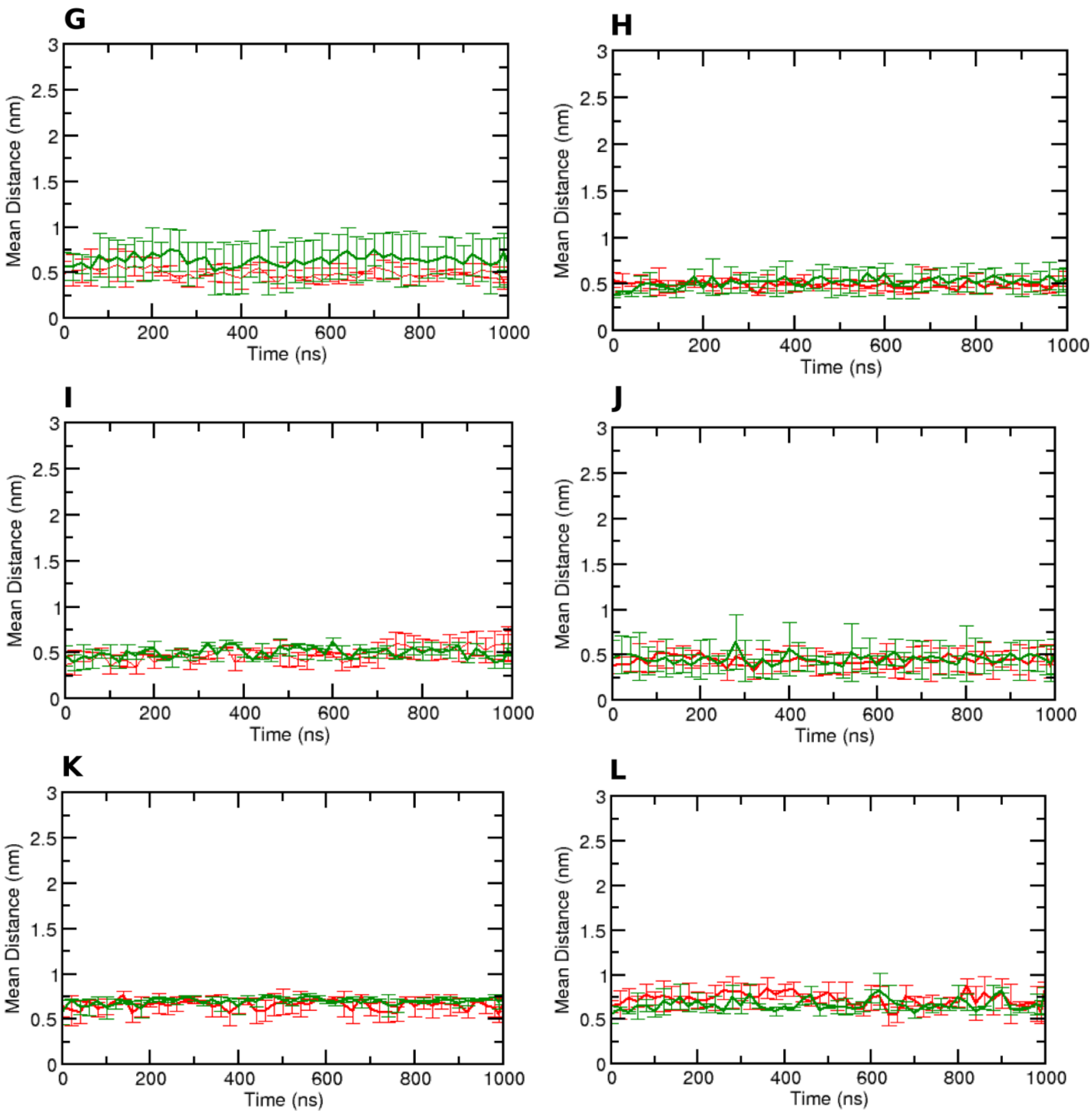
G
H
I
J
K
L
Mean Distance (nm)
Time (ns)
0
0.5
1
1.5
2
2.5
3
0
200
400
600
800
1000

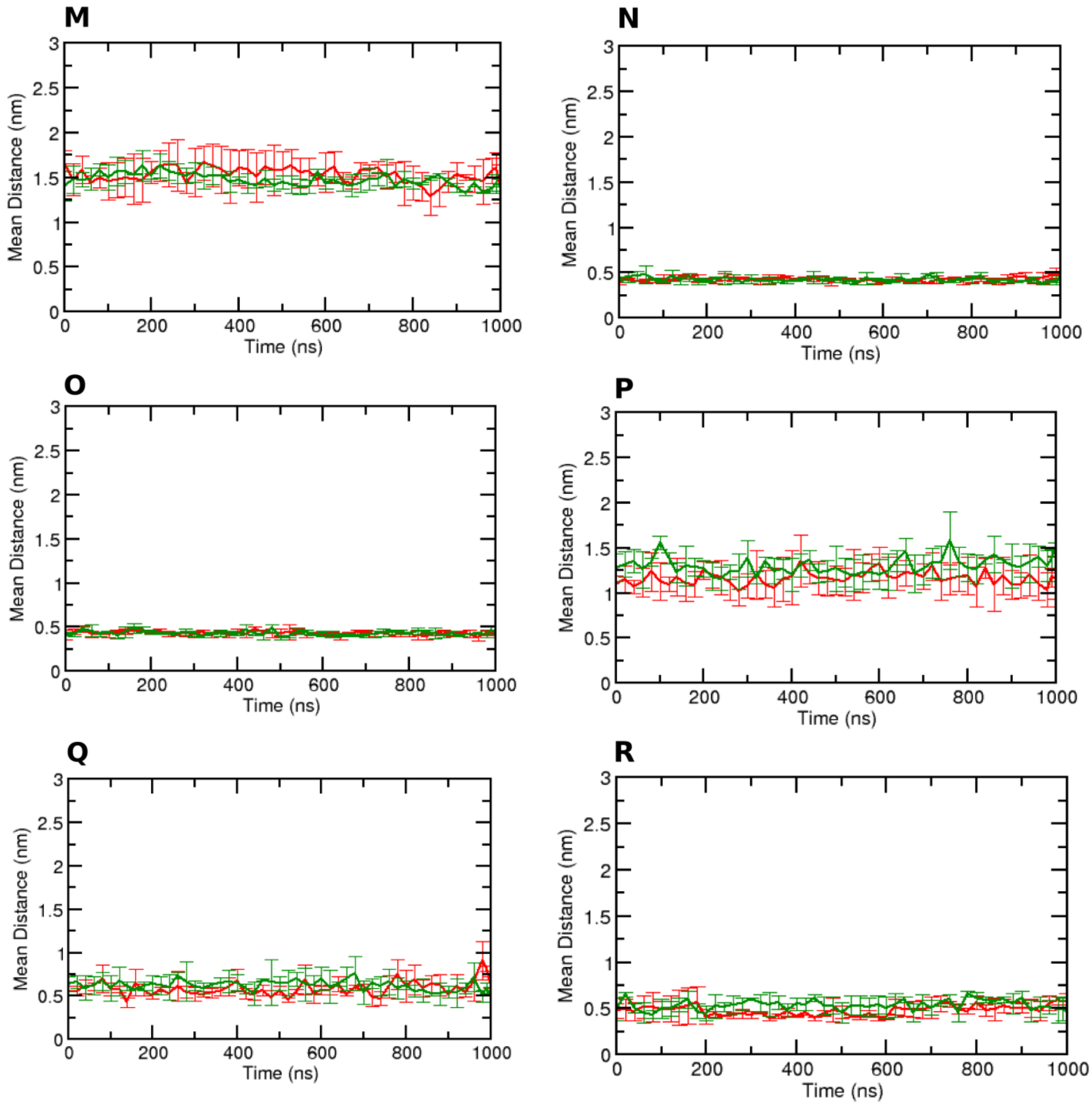
M
N
O
P
Q
R
Mean Distance (nm)
Time (ns)
0
0.5
1
1.5
2
2.5
3
0
200
400
600
800
1000

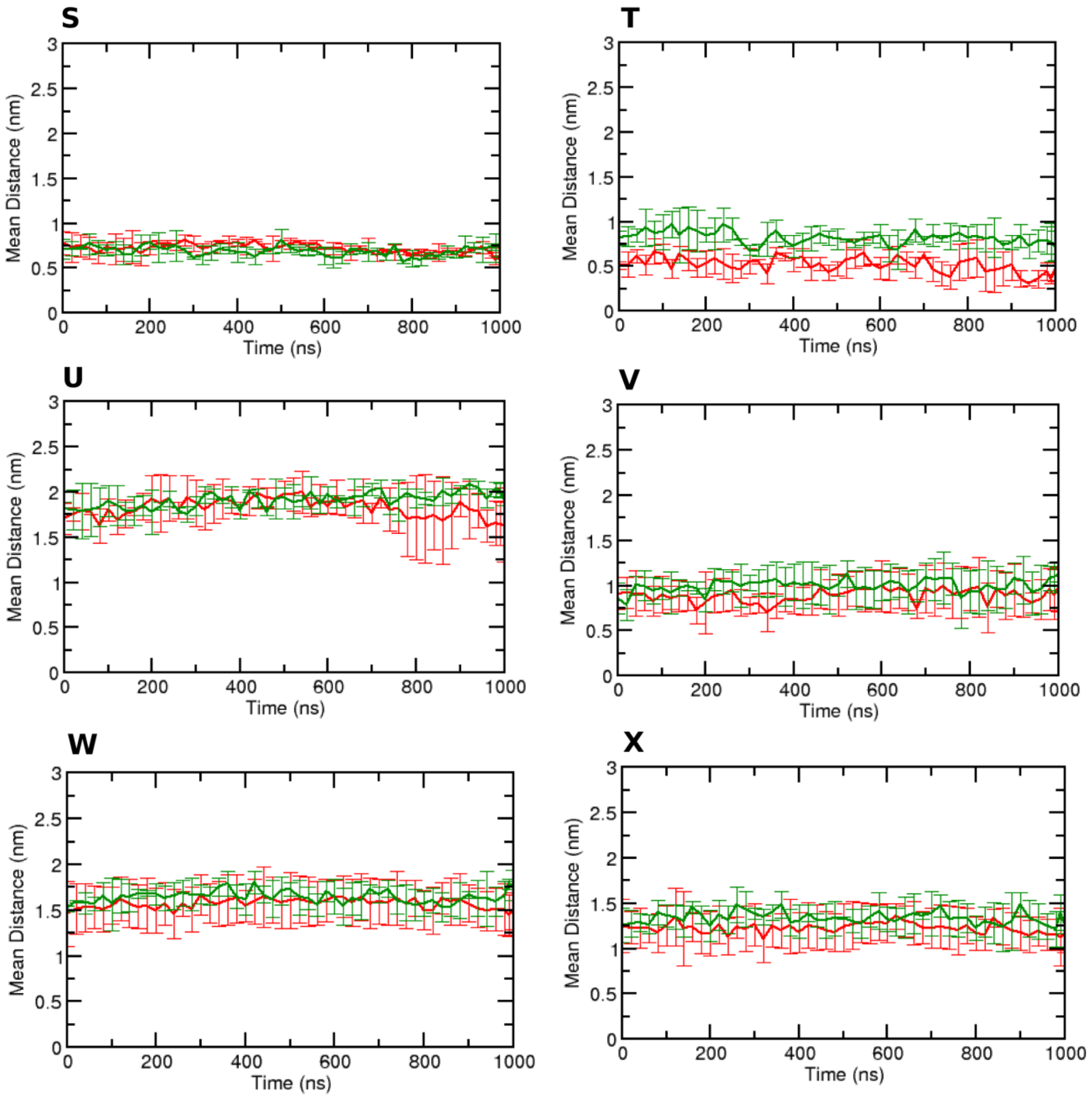

Supplementary Fig. 8: Distance between the side chains of the residue pairs (A) Ile47 & Gly320, (B) Gly50 & Pro323 (C) Asn51 & Ser319 (D) Val54 & Asn51 (E) Ile58 & Thr73 (F) Phe71 & Ile127 (G) Ile72 & Tyr326 (H) Ala76 & Val54 (I) Asp79 & Asn51 (J) Asp79 & Ser319 (K) Leu115 & Ser161 (L) Leu115 & Ser165 (M) Val117 & Met279 (N) Ala119 & Trp158 (O) Ala119 & Ser161 (P) Ile121 & Leu275 (Q) Cys125 & Met215 (R) Ala128 & Val218 (S) Tyr132 & Val218 (T) Tyr132 & Arg221 (U) Met215& Lys273 (V) Ile278 & Asn318 (W) Phe282 & Leu311 (X) Phe282 & Asn312. In each panel the red line indicates the distance for the Arg variant and the green line indicates the distance for the Gly variant respectively.
