# Supplementary material for: Molecular Insights into the Dynamics of Pharmacogenetically Important N-Terminal Variants of the Human β2-Adrenergic Receptor
Source: PLoS Comput Biol. 2014 Dec 11;10(12):e1004006. doi: 10.1371/journal.pcbi.1004006 (PMC4263363; doi:10.1371/journal.pcbi.1004006)
Supplement: S9 Figure — Characterization of additional homology models of the β2AR variants. Top-view snapshots of initial A) Arg model 2 B) Arg Model 3 C) Gly Model 2 D) Gly Model 3 chosen for 100 ns simulation. Panel E and F represent volumes (in Å3) of the non-occluded grid of vestibule 1 for the Arg and Gly variants, respectively. Panel G and H represent volumes (in Å3) of the non-occluded grid of vestibule 2 for the Arg and Gly variants, respectively. (I) Average distance between residues 289 and 203 for the Arg (red) and Gly (green) variants. (J) Average distance between residues 312 and 203 for the Arg (red) and Gly (green) variants. (PDF) [file pcbi.1004006.s009.pdf]

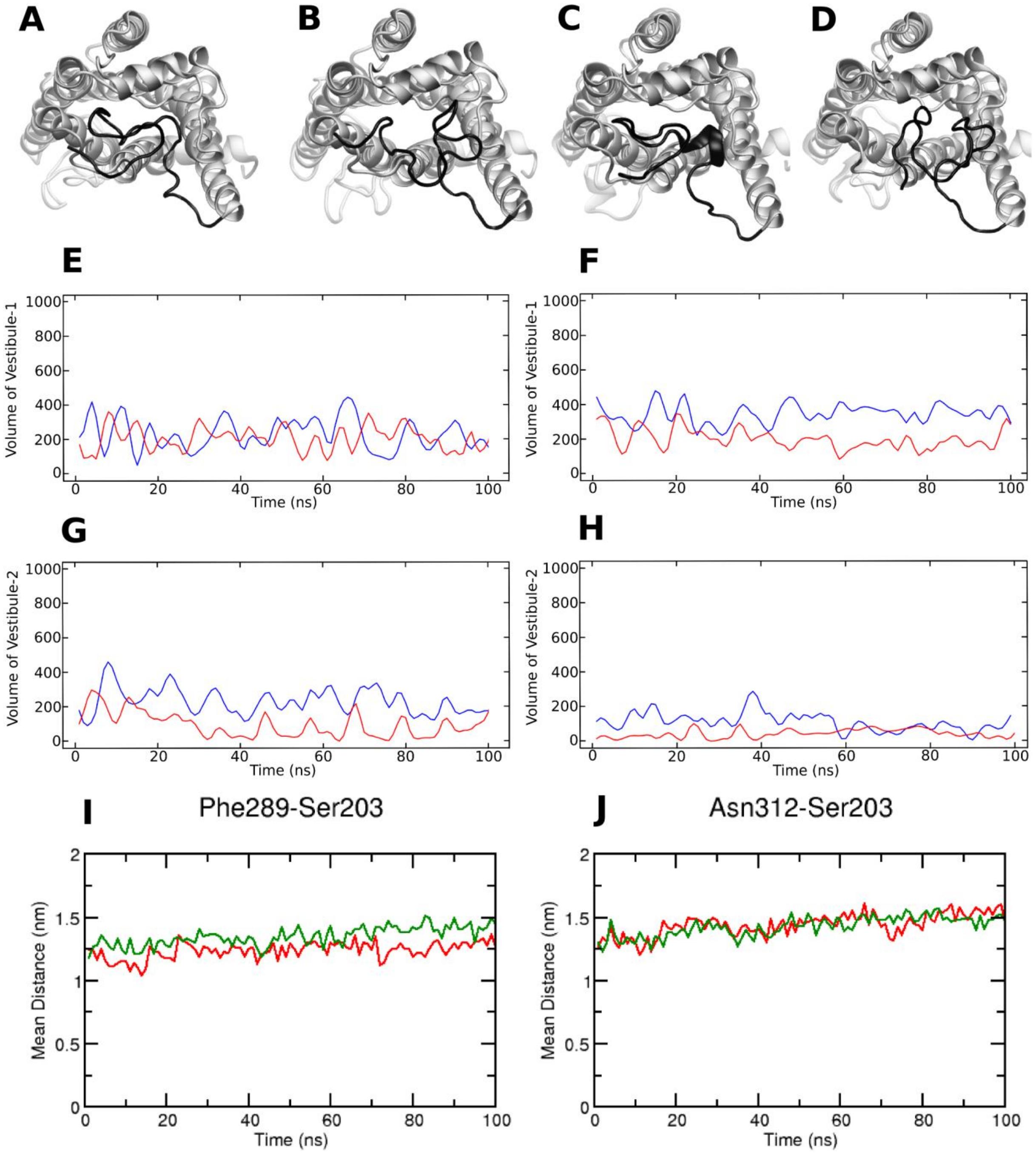


Supplementary Fig. 9: Top-view snapshots of initial A) Arg model 2 B) Arg Model 3 C) Gly Model 2 D) Gly Model 3 chosen for 100 ns simulation. Panel E and F represent volumes (in Å$^3$) of the non-occluded grid of vestibule 1 for the Arg and Gly variants, respectively. Panel G and H represent volumes (in Å$^3$) of the non-occluded grid of vestibule 2 for the Arg and Gly variants, respectively. (I) Average distance between residues 289 and 203 for the Arg (red) and Gly (green) variants. (J) Average distance between residues 312 and 203 for the Arg (red) and Gly (green) variants.
