# Supplementary material for: Molecular Insights into the Dynamics of Pharmacogenetically Important N-Terminal Variants of the Human β2-Adrenergic Receptor
Source: PLoS Comput Biol. 2014 Dec 11;10(12):e1004006. doi: 10.1371/journal.pcbi.1004006 (PMC4263363; doi:10.1371/journal.pcbi.1004006)
Supplement: S1 Table — Class A GPCRs from the GPCRDB which include full/partial N-terminal coordinates. (PDF) [file pcbi.1004006.s010.pdf]

Supplementary Table I: Class A GPCRs from the GPCRDB which include full /partial N-terminal coordinates.

Sr. No.	GPCR	PDB-ID	Resolution (Å)	Placement of N-terminal residues	Coverage (residues)	Ligand
1	Squid Rhodopsin	2Z73	2.50	top	9-358	Retinal
2	Squid Rhodopsin	2ZIY	3.70	top	4-373	Retinal
3	Native Opsin	3CAP	2.90	top	1-326	Retinal
4	5-HT1B	4IAQ	2.80	top	38-239 & 304-387	Serotonin
5	Neurotensin receptor NTS1	4GRV	2.80	top	52-268 & 300-379	Neurotensin
6	5-HT2B	4IB4	2.70	top	48-248 & 314-400	Serotonin
7	Bovine Rhodopsin	1U19	2.20	top	1-348	Retinal
8	NK1R	2KS9	NMR	top	2-364	Substance P
9	Bovine Rhodopsin	1F88	2.80	top	1-348	Retinal
10	Bovine Rhodopsin	1GZM	2.65	top	1-331	Retinal
11	Bovine Rhodopsin	1HZX	2.80	top	1-348	Retinal
12	Bovine Rhodopsin	1L9H	2.60	top	1-348	Retinal
13	Bathorhodopsin	2G87	2.60	top	1-348	Retinal
14	Lumirhodopsin	2HPY	2.80	top	1-348	Retinal
15	Ground state rhodopsin	2I35	3.80	top	1-326	Retinal
16	Ground state rhodopsin	2I36	4.10	top	1-326	Retinal
17	Photoactivated rhodopsin	2I37	4.15	top	1-333	Retinal
18	Rhodopsin	2J4Y	3.40	top	1-326	Retinal
19	NK1R	2KSA	NMR	top	2-363	Substance P
20	NK1R	2KSB	NMR	top	2-364	Substance P
21	9-cis-rhodopsin	2PED	2.95	top	1-348	Retinal
22	Rhodopsin mutant	2X72	3.00	top	1-326	Retinal
23	Squid Rhodopsin	3AYM	2.80	top	9-358	Retinal
24	Squid Isorhodopsin	3AYN	2.70	top	9-358	Retinal
25	Ground state bovine rhodopsin	3C9L	2.65	top	1-331	Retinal

26	Mutant bovine rhodopsin	3C9M	3.40	top	1-326	Retinal
27	Opsin	3DQB	3.20	top	1-326	Retinal
28	Bovine rhodopsin	3OAX	2.60	top	1-348	Retinal
29	CXCR4*	3ODU	2.50	top	27-901 & 230-328	SDF-1/CXCL12
30	CXCR4*	3OE0	2.90	top	25-228 & 231-303	SDF-1/CXCL12
31	Metarhodopsin-II	3PXO	3.00	top	1-326	Retinal
32	Metarhodopsin-II	3PQR	2.85	top	1-326	Retinal
33	Sphingosine-1 phosphate receptor 1	3V2Y	2.80	top	16-231 & 245-330	Sphingosine-1 phosphate
34	Sphingosine-1 phosphate receptor 1	3V2W	3.35	top	17-231 & 245-325	Sphingosine-1 phosphate
35	Rhodopsin mutant	4A4M	3.30	top	1-326	Retinal
36	G90D rhodopsin	4BEY	2.90	top	1-326	Retinal
