# Supplementary material for: Molecular Insights into the Dynamics of Pharmacogenetically Important N-Terminal Variants of the Human β2-Adrenergic Receptor
Source: PLoS Comput Biol. 2014 Dec 11;10(12):e1004006. doi: 10.1371/journal.pcbi.1004006 (PMC4263363; doi:10.1371/journal.pcbi.1004006)
Supplement: S2 Table — Contacts of the N-terminal residues with the rest of the receptor that are within 0.3 nm for at least 30% of simulation time. (PDF) [file pcbi.1004006.s011.pdf]

Supplementary Table II: Contacts of the N-terminal residues with the rest of the receptor that are within 0.3 nm for at least 30% of simulation time

Sr. No.	Residues	ARG variant			GLY variant		
		Simulation1	Simulation2	Simulation3	Simulation1	Simulation2	Simulation3
1	MET					304 ARG - 30.0 %	
2	GLY						
3	GLN						304 ARG - 40.9 %
4	PRO						
5	GLY				301 ASN – 95.0 %		304 ARG - 64.6 %<br>301 ASN - 32.6 %
6	ASN				194 PHE - 49.2 %<br>180 GLU - 82.9 %<br>305 LYS - 82.7 %<br>192 ASP - 93.1 %	180 GLU - 35.2 %<br>178 HIS - 37.6 %<br>181 ALA – 39.9 %<br>194 PHE – 46.2 %	192 ASP - 65.5 %
7	GLY				194 PHE - 59.3 %<br>296 HIS - 54.7 %<br>301 ASN - 79.1 %<br>180 GLU - 85.8 %	194 PHE - 60.8 %	192 ASP - 40.2 %<br>304 ARG - 55.6 %
8	SER				305 LYS - 86.8 %<br>301 ASN - 97.7 %	194 PHE - 50.4 %<br>300 ASP - 86.1 %	306 GLU - 42.0 %
9	ALA				304 ARG - 50.5 %<br>303 ILE – 39.3 %	301 ASN - 38.6 %	
10	PHE						
11	LEU				304 ARG - 38.4 %		
12	LEU						
13	ALA		301 ASN - 60.4 %				
14	PRO		305 LYS - 38.5 %				
15	ASN	300 ASP - 30.1 %	180 GLU - 76.7 %<br>301 ASN – 33.8 %	180 GLU - 72.7 %<br>305 LYS - 61.5 %		300 ASP - 70.2 %	
16	ARG/GLY		305 LYS - 35.0 %<br>194 PHE - 42.6 %<br>296 HIS - 42.2 %<br>308 TYR - 47.5 %<br>180 GLU - 58.1 %<br>193 PHE - 69.5 %	308 TYR - 36.3 %<br>296 HIS - 70.7 %<br>301 ASN - 92.8 %<br>180 GLU - 99.3 %			
17	SER		194 PHE - 65.1 %<br>180 GLU - 69.5 %	305 LYS - 68.9 %<br>180 GLU - 93.1 %	97 LYS - 64.9 %<br>189 THR - 57.9 %<br>191 CYS - 77.9 %<br>192 ASP - 90.0 %<br>98 MET – 31.9%	180 GLU - 61.0 %	
18	HIS	178 HIS - 54.8 %<br>194 PHE - 73.2 %<br>192 ASP - 91.6 %<br>185 TYR – 32.1 %	305 LYS – 33.6 %<br>192 ASP – 86.8 %	305 LYS - 50.8 %<br>194 PHE - 31.8 %<br>192 ASP - 42.6 %<br>312 ASN - 82.5 %<br>289 PHE - 79.5 %<br>308 TYR - 84.5 %	93 HIS - 40.5 %<br>308 TYR - 64.8 %<br>97 LYS - 70.6 %<br>305 LYS - 78.9 %<br>192 ASP - 92.7 %		192 ASP - 42.1 %<br>99 TRP - 53.5 %
19	ALA			305 LYS - 52.5 %			
20	PRO						
21	ASP		97 LYS - 91.0 %<br>189 THR – 32.1 %		304 ARG - 52.3 %<br>306 GLU - 77.1 %		
22	HIS		187 ASN - 48.7 %<br>188 GLU - 60.3 %	97 LYS - 39.7 %<br>188 GLU – 32.2 %		304 ARG – 30.9 %	
23	ASP	99 TRP - 52.5 %<br>305 LYS – 39.0 %	97 LYS - 65.3 %			304 ARG - 58.2 %<br>97 LYS - 94.4 %<br>306 GLU - 95.5 %<br>305 LYS - 98.0 %	97 LYS - 78.5 %<br>99 TRP – 36.8 %
24	VAL					97 LYS - 32.6 %	97 LYS - 51.4 %
25	THR	99 TRP - 48.1 %				306 GLU - 99.6 %	306 GLU - 99.9 %
26	GLN					306 GLU - 99.6 %	306 GLU - 74.5 %<br>32 TRP - 54 %
27	GLU		32 TRP - 75.1 %<br>97 LYS - 73.2 %	97 LYS - 38.5 %			
28	ARG	98 MET - 33.4 %<br>94 ILE - 66.7 %<br>99 TRP - 72.8 %<br>93 HIS - 72.1 %<br>96 MET - 76.6 %<br>97 LYS - 96.9 %	95 LEU - 32.5 %	94 ILE - 41.9 %<br>95 LEU - 71.4 %<br>32 TRP – 36.2 %		31 VAL - 34.4 %<br>32 TRP - 83.4 %	32 TRP - 66.1 %

Shahane et al, Molecular insights into the dynamics of pharmacogenetically important N-terminal variants of the human β_2-adrenergic receptor
