# Supplementary material for: Molecular Insights into the Dynamics of Pharmacogenetically Important N-Terminal Variants of the Human β2-Adrenergic Receptor
Source: PLoS Comput Biol. 2014 Dec 11;10(12):e1004006. doi: 10.1371/journal.pcbi.1004006 (PMC4263363; doi:10.1371/journal.pcbi.1004006)
Supplement: S3 Table — Templates used to model the N-terminal region of human β2AR variants. (PDF) [file pcbi.1004006.s012.pdf]

Supplementary Table III: Templates used to model the N-terminal region of human β_2AR variants

**Target Region**	**Name of template**	**PDB ID**	**Resolution (Å)**	**Length of the N-terminal region**	**% Similarity with the N-terminal region of β_2AR (Gly variant) Performed by Needle algorithm at EBI**
Residue 1-28	Bovine rhodopsin	1U19	2.2	33 residues	13%
Residue 1-28	Squid rhodopsin	2ZIY	3.7	30 residues	3.6%
Residue 1-28	NKR1(p-subunit)	2KS9	NMR	28 residues	3.8%
Residue 1-28	CCR	2L87	NMR	30 residues	1.8%
Residue 1-28	CXCR4	2K03: Chain B	NMR	38 residues	21.6%
